# Expression of Amyloid Precursor Protein, Caveolin-1, Alpha-, Beta-, and Gamma-Secretases in Penumbra Cells after Photothrombotic Stroke and Evaluation of Neuroprotective Effect of Secretase and Caveolin-1 Inhibitors

**DOI:** 10.3390/biomedicines10102655

**Published:** 2022-10-20

**Authors:** Svetlana Sharifulina, Andrey Khaitin, Valeria Guzenko, Yuliya Kalyuzhnaya, Valentina Dzreyan, Alexandr Logvinov, Natalia Dobaeva, Yan Li, Lei Chen, Bin He, Svetlana Demyanenko

**Affiliations:** 1 Laboratory of Molecular Neurobiology, Academy of Biology and Biotechnology, Southern Federal University, pr. Stachki 194/1, 344090 Rostov-on-Don, Russia; 2 Department of General and Clinical Biochemistry no.2, Rostov State Medical University, st. Nakhichevansky 29, 344000 Rostov-on-Don, Russia; 3 School of Pharmacy, Guizhou Medical University, Guiyang 550004, China

**Keywords:** amyloid precursor protein, photothrombotic stroke, ischemia, alpha-secretase, beta-secretase, gamma-secretase, caveolin-1, apoptosis, astrocyte activation

## Abstract

Our studies reveal changes in the expression of the main participants in the processing of amyloid precursor protein (APP) in neurons and astrocytes after photothrombotic stroke (PTS). Here we show the increase in the level of N- and C-terminal fragments of APP in the cytoplasm of ischemic penumbra cells at 24 h after PTS and their co-immunoprecipitation with caveolin-1. The ADAM10 α-secretase level decreased in the rat brain cortex on the first day after PTS. Levels of γ-secretase complex proteins presenilin-1 and nicastrin were increased in astrocytes, but not in neurons, in the penumbra after PTS. Inhibitory analysis showed that these changes lead to neuronal death and activation of astrocytes in the early recovery period after PTS. The caveolin-1 inhibitor daidzein shifted APP processing towards Aβ synthesis, which caused astroglial activation. γ-secretase inhibitor DAPT down-regulated glial fibrillary acidic protein (GFAP) in astrocytes, prevented mouse cerebral cortex cells from PTS-induced apoptosis, and reduced the infarction volume. Thus, new generation γ-secretase inhibitors may be considered as potential agents for the treatment of stroke.

## 1. Introduction

Stroke is the second leading cause of mortality and the major cause of physical disability in adults worldwide [1]. Preclinical testing of potential drugs has not yet found agents without serious side effects, limiting the propagation of pathological processes from the primary site of damage to surrounding healthy tissues, and protecting nerve cells [2,3]. Thus, deep and comprehensive studies on molecular mechanisms of the pathological processes after stroke are required.

Our previous proteomic studies have shown that the expression of amyloid precursor protein (APP), as well as nicastrin and apolipoprotein E (APOER or LRP1), involved in the processing and functioning of APP, increased in the penumbra at 1–4 h after acute photothrombotic exposure; hence, we decided to study APP and its proteolytic system understroke [4].

The APP protein has been intensively studied since the 1980s due to its central role in the development of Alzheimer’s disease (AD). Its fragment β-amyloid peptide (Aβ) accumulates in amyloid plaques in the brain of AD patients. APP is an evolutionarily conserved protein [5,6]. A high level of APP expression in the brain indicates its important role in the nervous system. It is involved in the development, differentiation, and function of neurons, neurite growth, synapse and long-term memory formation, brain integrity maintenance, and neuronal response to damage [7,8]. However, the specific biochemical and physiological functions of APP and its proteolytic products are still unknown. The accumulation of APP in damaged neurons after ischemic stroke indicates its important role in stroke-induced pathological processes in the nerve tissue [9,10].

APP is a large transmembrane glycoprotein that crosses the plasma membrane (PM) once. Its large N-terminal domain faces the extracellular environment, while its small C-terminal domain faces the cytoplasm. APP undergoes proteolytic cleavage by α-, β-, and γ-secretases to form several peptides: sAPPα (soluble amyloid precursor protein alpha), sAPPβ (soluble amyloid precursor protein beta), Aβ (β-amyloid peptide), AICD (amyloid precursor protein intracellular domain), and some less studied peptides. APP proteolytic products have independent activity and are involved in various cellular processes. There are amyloidogenic and non-amyloidogenic pathways of APP proteolysis.

In the non-amyloidogenic pathway, α-secretase cleaves a large N-terminal fragment of sAPPα from APP. At the same time, Aβ (the APP fragment that is immersed in the membrane) is cleaved and inactivated. Then γ-secretase cleaves off the intracellular AICD peptide that degrades in the cytoplasm. Cleavage of APP by α-secretase occurs mainly on the cell surface, although some part of APP is cleaved along the pathways of processing and traffic in cisterns and vesicles of the endoplasmic reticulum and Golgi apparatus [11,12]. Proteinases of the ADAM (a disintegrin and metalloproteinase) family act as α-secretase in mammalian cells and ADAM10 protein plays the main role in APP processing in the non-amyloidogenic pathway [13,14]. Amyloidogenic processing of APP occurs in specialized regions of the cell membranes, the lipid rafts. However, ADAM10-mediated non-amyloidogenic processing is believed to occur in the non-raft region of the membrane [15,16,17].

β-secretase is an aspartyl protease represented by two main isoforms: BACE1 (beta-site APP cleaving enzyme 1) and BACE2. BACE1 is abundant in the nervous system (in neurons, astrocytes, and oligodendrocytes) while BACE2 is abundant in peripheral tissues (melanocytes or pancreatic β-cells). 

In the amyloidogenic pathway, BACE1 and γ-secretase cleave APP in the plasma membrane so that the Aβ peptide is released into the environment and AICD is released into the cytoplasm. During cerebral ischemia, BACE1 is activated as a result of oxidative stress and stimulation of the oxygen sensor HIF1α [18,19]. γ-secretase is a large multi-subunit enzyme consisting of presenilin-1 (PS1) that performs a proteolytic function, presenilin-2 (presenilin enhancer 2, PEN-2) that associates and causes the endoproteolysis of PS1 into the N-terminal fragment (NTF) and C-terminal fragment (CTF), involved in the substrate recognition of nicastrin (NCT), and the anterior pharynx-defective 1 (APH-1) protein that forms a platform for subunit binding [20]. The results of γ-secretase activity include the release of the amyloid peptide Aβ into the extracellular environment promoting the development of AD and the release of the rest into the cytoplasm as the transcription factor AICD is regulating the expression of proapoptotic genes [11]. The subcellular localization of APP influences its proteolytic processing and Aβ formation, not only the localization of β- and γ-secretases in lipid rafts and their co-localization with caveolin-1 but also the exclusion of ADAM10 from lipid rafts lead to Aβ formation [16]. On the other hand, non-amyloidogenic APP processing occurs on the cell surface, where α-secretase is localized while amyloidogenic APP processing occurs after the internalization of APP from the cell surface via endocytosis and only partially on the cell surface [21,22]. Caveolin-1 positively regulates APP cleavage by α-secretase [23] and, vice versa, down-regulates BACE1 activity [24], suggesting that caveolae and caveolins may play a key role in APP proteolysis. Caveolin-1 level significantly increases in neurons with aging [25,26] and under oxidative stress [27].

In this study, we investigated the expression and localization of APP in rat brain cells after photothrombotic stroke (PTS). We studied the expression and localization of α-secretase ADAM10, β-secretase BACE1, and components of the γ-secretase complex presenilin 1 and nicastrin involved in APP proteolysis in rat brain neurons and glial cells after PTS. The effect of ischemia on caveolin-1 levels and co-immunoprecipitation of caveolin-1 with N- or C-terminal fragments of the APP (N-APP and C-APP) and ADAM10 was also investigated. In addition, we studied the effect of secretase inhibitors and caveolin-1 inhibitors on apoptosis, expression of the glial fibrillary acidic protein (GFAP) in astrocytes, and the volume of mouse brain infarction after PTS.

## 2. Methods

### 2.1. Antibodies 

For Western blot analysis and immunofluorescence microscopy, rabbit antibodies were used: anti-ADAM-10, C-terminus (A2726), anti-BACE1 (SAB2100200), anti-nicastrin (ab1) (PRS3983), anti-presenilin-1 (PRS4203), anti-caveolin-1 (marker of lipid raft) (A19006); mouse antibodies NeuN (marker of neurons) (MAB377), GFAP (marker of astrocytes) (SAB5201104), and anti-caveolin-1 (marker of lipid raft) (SAB4200216). To determine the expression and localization of the APP protein by Western blot analysis, immunofluorescence microscopy and immunoelectron microscopy in nerve cells, we used antibodies that specifically recognize the N- or C-terminal fragments of the APP protein (N-APP and C-APP). According to the manufacturer (Merck, Darmstadt, Germany), the anti-rabbit N-APP antibody (SAB4200536) recognizes the N-terminal extracellular domain of human, rat, or mouse APP, and its proteolytic products sAPPα. The anti-rabbit C-APP antibody (A8717) is specific for the sequence of amino acids 676–695 at the C-terminal of APP and anti-β-Amyloid antibody (A8354).

All antibodies and reagents used were purchased from the Moscow branch of Merck (Merck Life Science LLC, Moscow, Russian Federation), except for rabbit antibodies anti-caveolin-1 (A19006) from ABclonal (Woburn, MA, USA).

### 2.2. Animals

The experiments were carried out on adult male rats (3–4 months, 200–250 g). The experiments with inhibitors were performed on male mice of the outbred CD-1 stock (14–15 weeks old, 20–25 g). Outbred Wistar rats and CD-1 mice were purchased from a farm in Pushchino, Moscow Region (http://www.spf-animals.ru/animals/rats/, accessed on 13 May 2020). Outbred white mice and rats were obtained from the vivarium of the Rostov Scientific Research Institute of Microbiology and Parasitology. Animals were kept in standard cages in groups of 4–5 animals with free access to food and water under standard conditions: 12 h light/12 h dark cycle, 22–25 °C, air exchange rate 18 changes per hour. International, national, and institutional guidelines for the care and use of animals were followed. All experimental procedures were carried out in accordance with European Union directives 86/609/EEC on the use of experimental animals and local legislation on the ethics of animal experimentation. Animal protocols were evaluated and approved by the Animal Care and Use Committee of the Southern Federal University (Permit No. 08/2016). For the entire period of detention and before the experiment the animals were properly cared for with daily veterinary examination (body position in space, activity), thermometry, and weighing of each one. The adequate depth of anesthesia was achieved in about 30 min. The depth of anesthesia was assessed by the absence of a plantar reflex and a reaction to pinching the membrane between the fingers), a decrease in or absence of muscle tone in the limbs, and a slow regular heart rate and respiratory rhythm. The following measures of physiological support of an animal during anesthesia and experimental procedures were obtained: prevention of dry eyes and damage to the cornea by placing an ophthalmic ointment in the conjunctival sac and temperature maintenance via an electrically heated mat. After the surgical intervention, each animal was placed into a separate warm clean cage until the complete recovery from anesthesia. Further postsurgical care included the administration of analgesics, antimicrobials, daily monitoring of the animals’ state for signs of pain and distress; special attention was paid to the condition of sutures and the irradiation area.

### 2.3. Photothrombotic Stroke Model

For a model of ischemic stroke, we used unilateral photothrombotic stroke (PTS) in the somatosensory cerebral cortex of rats or mice. In PTS, local laser irradiation induces photoexcitation of the introduced photosensitizing dye Bengal rose. Due to its physical properties, it does not penetrate cells and remains in blood vessels. After laser irradiation, highly reactive singlet oxygen is generated and damages the vascular endothelium, causing platelet aggregation and vascular thrombosis [28].

Experiments were performed as described before [29]. Briefly, rats were anesthetized with intraperitoneal injections of telazol (50 mg/kg) and xylazine (10 mg/kg). The animals were fixed, the periosteum was removed, and a longitudinal incision was made in the skull skin. Rose Bengal (20 mg/kg) (R4507, Merck, Moscow, Russia) was injected into the subclavian vein. Then the somatosensory cortex (3 mm lateral to the bregma) was irradiated through the relatively transparent cranial bone with a diode laser (532 nm, 60 mW/cm^2^, Ø3 mm, 30 min). This mode of exposure induces the formation of an infarction core with a diameter of about 3 mm surrounded by a penumbra about 1.5 mm wide [30]. After anesthesia, the rats were decapitated in 4 h or 24 h or 7 days after PTS. The brain was removed and a section of the cortex corresponding to the infarction core was removed on ice with a cylindrical knife (Ø 3 mm) and then a 2-mm ring was cut around the irradiation zone with another knife (Ø 7 mm), approximately, corresponding to the penumbra tissue (experimental sample, respectively, PTS4, PTS24, and PTS7d). The control groups included sham-operated (SO) animals subjected to the same procedures but without the photosensitizer administration. The obtained tissue samples were further used for Western blot analysis.

Experiments with inhibitors of α-, β-, and γ-secretases were carried out on mice. Mice were anesthetized at 25 mg/kg telazol and 5 mg/kg xylazine. Rose Bengal at a concentration of 15 mg/mL was administered intraperitoneally at a dose of 10 μL/g of body weight. At 5 min after the photosensitizer administration, the area of the mouse skull free from the periosteum was irradiated with a diode laser in the sensorimotor cortex area (2 mm lateral to the bregma). Irradiation parameters: wavelength 532 nm, intensity 0.2 W/cm^2^, beam diameter 1 mm, duration 15 min. The control groups included SO animals subjected to the same operations but without the photosensitizer administration. At 3, 7, and 14 days after laser irradiation, the mice were decapitated and the brain was removed to study the extent of damage, the level of apoptosis of cells in the perifocal region and the expression of the GFAP protein in astrocytes. The surgery is non-invasive with 100% survival of animals before the decapitation.

### 2.4. Cytoplasmic and Nuclear Fractions of Brain Tissue Extraction

Cytoplasmic and nuclear fractions were obtained using the CelLytic™ NuCLEAR™ Extraction Kit (NXTRACT, Sigma-Aldrich, Darmstadt, Germany). To do this, the samples were homogenized on ice for 3 min using a Vibra-Cell VCX 130 ultrasonic homogenizer (Sonics, Newtown, CT, USA) in Lysis Buffer, which is included in the CelLytic™ NuCLEAR™ Extraction Kit, supplemented with a mixture of inhibitors, proteases, and phosphatases (PPC1010, Sigma-Aldrich, Darmstadt, Germany), necessary for the preservation of proteins and their phosphorylated forms, as well as nuclease benzonase (E1014, Sigma-Aldrich, Darmstadt, Germany), which destroys nucleic acids. After the homogenization, the samples were centrifuged for 20 min at 10,000–11,000× *g* at 4 °C in a Mikro 220 R centrifuge (Hettich, Tuttlingen, Germany). Then, the supernatant containing cytoplasmic proteins was collected and nuclear proteins were extracted from the sediment containing cell fragments and cell nuclei using the Nuclear Extraction Buffer included in the NXTRACT Reagent Kit. To carry out this process, the pellet was resuspended and incubated for 40 min with this buffer. The lysate was centrifuged for 5 min at 20,000–21,000× g at 4 °C.

In the resulting supernatant, containing nuclear proteins and the previously obtained cytoplasmic fraction, the protein content was determined using the Bradford reagent (B6916, Sigma-Aldrich). The lysates were then aliquoted, frozen in liquid nitrogen, and stored at −80 °C for further Western blot analysis.

The purity of the fractions was checked as follows: negative control of the cytoplasmic marker in the nuclear fraction was used and, vice versa, negative control of the nuclear marker in the cytoplasmic fraction was used as well. The acetylated histone protein H4 (ac-H4) was used as a nuclear fraction marker. We used an H4 anti-acetyl-Histone antibody obtained from rabbits (No. 06-866, Merck), diluted at 1:500. Proteinglyceraldehyde-3-phosphate dehydrogenase (GAPDH) was used as a marker of a cytoplasmic fraction. We used an anti-GAPDH antibody obtained from rabbits (G9545, Sigma-Aldrich) at a 1:1000 dilution. The cytoplasmic fraction was confirmed by the absence of ac-H4, and the nuclear fraction was confirmed by the absence of the cytoplasmic fraction marker GAPDH (Figure S1).

### 2.5. Immunoblotting

Expression of C-APP, N-APP, Aβ, ADAM10, BACE1, presenilin 1, nicastrin, and caveolin-1 in the cytoplasmic fraction of rat cerebral cortex cells after PTS was studied using the Western blot method as described previously [20]. Briefly, the rat cortical tissue samples were homogenized on ice using a Vibra-Cell VCX 130 ultrasonic homogenizer. Nuclear and cytoplasmic fractions were isolated using the CelLytic NuCLEAR Nuclear Fraction Extraction Kit.

Samples containing 10–20 µg of protein per 15 µL were subjected to electrophoretic separation in a polyacrylamide gel (7–10%) in the presence of sodium dodecyl sulfate in a mini-PROTEAN Tetra cell (Bio-Rad, Hercules, CA, USA). ColorBurst Electrophoresis Marker (C1992, Sigma-Aldrich) was used as a standard protein marker. After the separation, the proteins were subjected to electrophoresis onto a PVDF membrane (polyvinyl difluoride membrane 162-0177, Bio-Rad) using the Trans-Blot^®^ Turbo Transfer System (Bio-Rad, USA). After washing with PBS, the membrane was successively incubated for one hour in blocking buffer (TBS 1% Casein Blocker, Bio-Rad) and overnight at 4 °C with primary rabbit anti-C-APP (A8717, Merck, 1:500) or anti-N-APP (SAB4200536, Merck, 1:500) antibodies; anti-ADAM-10, C-terminus (A2726); anti-BACE1 (SAB2100200); anti-nicastrin (ab1) (PRS3983); anti-presenilin-1 (PRS4203); anti-caveolin-1 (A19006, ABclonal, 1:500); mouse anti-β-actin antibodies (A5441, 1:5000), mouse monoclonal anti-β-Amyloid antibody (A8354, 1:500) (Table S1 Supplementary).

After the incubation, the membranes were washed in Tris buffer with the addition of 0.1% Tween-20 (TTVS, 10 mM; pH 8) and incubated for one hour at room temperature with a secondary anti-rabbit antibody IgG peroxidase (A6154, Merck, 1:1000). Protein detection was performed on Clarity Western ECL Substrate (Bio-Rad). Chemiluminescence was analyzed using the Fusion SL gel documentation system (Vilber Lourmat, Collégien, France). The obtained images were processed using the VisionCapt software package (https://visioncapt.software.informer.com/, accessed on 20 August, 2020).

### 2.6. Co-Immunoprecipitation

Co-immunoprecipitation (Co-IP) was performed to confirm the fact of protein–protein interaction between C-APP, N-APP, ADAM-10, and caveolin-1. Co-immunoprecipitation was performed according to the Sileks commercial kit (Sileks, Moscow, Russia) using magnetic particles with protein G (SileksMag-Protein G, cat. no. K0182) in accordance with the manufacturer’s recommendations. For this purpose, in a cytoplasmic protein extract of penumbra tissue, obtained 24 h after photothrombotic exposure (PTS24), endogenous caveolin-1 proteins were immunoprecipitated with anti-caveolin-1 antibody and co-precipitated C-APP, N-APP, or ADAM-10 were subsequently detected by antibodies against respective proteins. To visualize the protein–protein interaction, the resulting immunoprecipitate was subjected to immunoblotting.

The primary antibodies: rabbit anti-C-APP (A8717, 1:500), anti-N-APP (SAB4200536, 1:500), anti-ADAM-10 (A2726, 1:500) caveolin-1 (SAB4200216, 1:500) were used. The expression level of the caveolin-1 protein served as a Co-IP control. HRP-conjugated antibodies (goat anti-rabbit IgG-HRP, Merk A6154, 1:1000; goat anti-mouse IgG-HRP, Amersham NIF825, 1:1000) were used as secondary antibodies. Proteins were identified in immunoblotting. 

### 2.7. Immunofluorescence Microscopy

The double immunofluorescence method was used to evaluate the expression and distribution of α-, β- and γ-secretases, and caveolin-1 in penumbra neurons and astrocytes in rats at 4 and 24 h, and 7 days after PTS. The isolated rat brain was fixed in 4% paraformaldehyde overnight and placed in a 30% sucrose solution. Frontal 20 μm thick brain slices (+4.5 mm to −2.5 mm from bregma), obtained using a Leica VT1000 S vibratome (Leica Biosystems, Deer Park, IL, USA), were washed in PBS and incubated in 5% bovine serum albumin c 0.3% TritonX-100 in PBS for one hour at room temperature and then incubated overnight at 4 °C in the same BSA solution with antibodies added: anti-C-APP (1:500), anti-N-APP (1:500), anti-ADAM10 (1:500), anti-BACE1 (1:500), anti-nicastrin (ab1) (1:500), anti-presenilin-1 (1:500), and anti-caveolin-1 (A19006, ABclonal, 1:500), anti-Caspase 3, active (C8487, 1:500), as well as antibodies to NeuN (neuron marker) (1:1000) and GFAP (astrocyte marker) (1:1000), and then with fluorescent secondary anti-rabbit CF488A (SAB4600045, 1:1000) or anti-mouse CF555 (SAB4600302, 1:1000) antibodies. Hoechst 33342 was used as a marker of cell nuclei. After washing in PBS, slices were incubated for one hour with fluorescent secondary anti-rabbit CF488A (SAB4600045, 1:1000) or anti-mouse antibodies CF555 (SAB4600302, 1:1000). The slices were then mounted on glass slides in 60% glycerol/PBS. The results were analyzed using Nikon Eclipse FN1 fluorescent microscope equipped with a Nikon Digital Sight DS-5Mc digital camera (Nikon, Tokyo, Japan) with NIS Elements and Olympus BX51 microscope equipped with an OrcaFlash 4.0 V3 digital camera with HCImage Live software (Hamamatsu, Hamamatsu City, Japan).

Quantitative evaluation of the fluorescence of the experimental and control preparations was carried out on 10–15 images obtained with the same digital camera settings. To isolate and calculate the fluorescence intensity, we used the “Threshold’’ method of the Adjust menu in the ImageJ application (http://rsb.info.nih.gov/ij/, NIH, USA, accessed on 20 October 2021). For better isolation, cells were cut off background pixels using the Subtract background feature in the Process menu. Next, using the capabilities of the Analyze Particles and ROI Manager menus, cells were isolated and their total fluorescence intensity was measured. The data were normalized after background subtraction:

$$I=\frac{I_{m}-I_{b}}{I_{b}},$$

where *I_m_* is the average cell fluorescence intensity and *I_b_* is the average background fluorescence outside the cells. Protein co-localization was assessed using the ImageJ application with the JACoP plugin. The co-localization coefficient M1 represents the proportion of pixels in the green channels relative to the total signal recorded in the red channel (marker).

### 2.8. Inhibitor Assay

Batimastat (batimastat (BB-94); SML0041) was used as an α-secretase inhibitor; LY2886721 (SML3013), as a β-secretase inhibitor); DAPT (D5942), as an inhibitor of γ-secretases. 

Batimastat was dissolved in DMSO and administered intraperitoneally to CD-1 mice at a dose of 50 mg/kg (or 3 mg/mL) one hour after irradiation for five days. Batimastat was previously shown to efficiently penetrate the brain when administered intraperitoneally [31].

LY2886721 is a potent and selective active site inhibitor of β-secretase (BACE1,2) without inhibition of other proteases such as cathepsin D, pepsin, and renin [32]. The LY2886721 preparation was dissolved in 6.7% DMSO and 5% Tween 20 in PBS and administered to animals intraperitoneally at a dose of 10 mg/kg/day for five days. 

DAPT, a γ-secretase inhibitor, was dissolved in 5% DMSO and administered to animals intraperitoneally at a dose of 10 mg/kg/day for five days [33].

Daidzein (Sigma-Aldrich, 486-66-8), a caveolin-1 inhibitor was dissolved 1:10 in a solution of dimethyl sulfoxide:phosphate-buffered saline (pH 7.2) and administered at 0.4 mg/kg/day, subcutaneously from 1st to 14th day after PTS [27].

### 2.9. Assessment of the Cerebral Cortex Infarction Volume in Mice after PTS

To assess the infarction volume, brain slices of mice were stained with 2,3,5-triphenyltetrazolium chloride (TTX; T8877, Sigma) at 3, 7, and 14 days after PTS. After decapitation, the brain was quickly removed and placed in a pre-chilled brain matrix of adult mice (J&K Seiko Electronic Co., Ltd.). The matrix with brain tissue was transferred to a freezer (−80 °C) for 3–5 min and cut into 2 mm thick sections. These sections were stained with 1% TTX for 30 min in the dark at 37 °C. Using the ImageJ image analysis application (http://rsb.info.nih.gov/ij/), the areas of infarction zones on each slice were measured, summed, and multiplied by the slice thickness (2 mm).

### 2.10. Estimation of the Number of Apoptotic Cells

Apoptotic cells were visualized using the TUNEL (TdT-mediated dUTP-X nick-end labeling) method which marks DNA strand breaks using the In Situ Cell Death Detection Kit, TMR red (no. 12156792910, Roche). At 3, 7, and 14 days after PTS and administration of inhibitors, mice were decapitated and frontal sections of the brain 20 μm thick were made on Leica VT 1000 S vibratome (Germany). The sections were treated with the reagents of the kit according to the manufacturer’s recommendations with the addition of Hoechst 33342 at a final concentration of 10 µg/mL and incubated for one hour at 37 °C.

The apoptotic index (AI) was calculated for TUNEL-positive cells (red fluorescence) in the perifocal region and the cortex of sham-operated animals within the whole area of the mount at a magnification of 20× using the formula: AI = (TUNEL-positive cell number)/(Total cell number (stained with Hoechst 33342)) × 100.

### 2.11. Electron Immunohistochemistry

Animal brain fixation was performed via transcranial perfusion under anesthesia (Nembutal at a dose of 60 mg/kg) using the Perfusion Two perfusion system(Leica Biosystems, Deer Park, IL, USA) equipped with an automatic pump. Perfusion was first carried out with a phosphate buffer solution, pH 7.4, brought to 37 °C (Merck, Darmstadt, Germany), and then with a cooled fixative solution, 4% paraformaldehyde (Merck, EMS, Kenilworth, NJ, USA) in phosphate buffer (pH 7.4). Then the brain was removed and placed in a fixative solution for additional fixation overnight at a temperature of 4 °C. After the post-fixation, a section was isolated from the brain along the coordinates: the first incision was 0.2 mm rostral from the bregma, the second incision was 6.04 mm caudal from the bregma while the brain was not dissected laterally. The excised brain fragment was glued at the caudal side of the cut down to the vibratome table VT 1000E (Leica Biosystems, Deer Park, IL, USA). Next frontal sections 60 µm thick were made.

Electronic immunohistochemistry was performed on rat brain slices according to the pre-embedding protocol. The pre-embedding method (before embedding) is based on the fact that the incubation of slices with primary and secondary antibodies as well as the detection of immune complexes takes place before wiring and embedding the sections in epoxy resin for electron microscopy. Vibratome slices of 60 μm were placed alternately in solutions of 6%, 15%, and 30% sucrose for cryoprotection. Unmasking of antigenic activity was carried out by instantaneous freezing of sections over vapors of liquid nitrogen and subsequent thawing in phosphate buffer. The slices were then incubated in primary anti-APP antibodies supplemented with 0.1% sodium azide to prevent bacterial growth. Incubation was carried out for four days at 20 °C. After washing in a phosphate buffer, the slices were incubated in secondary antibodies RTU Envision Flex/HRP anti-mouse, anti-rabbit (Dako, Glostrup, Denmark) for 24 h at 20 °C. Immune complexes were detected using the EnVision HRP + Peroxidase imaging system (Dako, Glostrup, Denmark). Then, tissue processing was carried out by standard methods for electron microscopic examination. After washing in a phosphate buffer for at least 15 min, the slices were additionally post-fixed in 1% OsO4 solution in the phosphate buffer for 1.5 h. Then all tissue samples were dehydrated in ascending alcohols and absolute ethanol, processed in three shifts of propylene oxide and embedded in an epoxy resin based on Epon-812. The slices were placed in a drop of resin between two glass slides coated with a water-soluble anti-adhesive Liquid Release Agent (EMS, USA). Polymerization of brain tissue was carried out at 70 °C overnight. Fragments of the studied zones were excised from the sections obtained after polymerization with a blade under a stereotaxic magnifying glass and polymerized to a prefabricated block of epoxy resin. Single and serial (up to 20 sections in one tape) 70 nm thick ultrathin slices were made using an EM UC 7 ultramicrotome (Leica, Germany) and an ultra 45° diamond knife (Diatome, Nidau, Switzerland), counterstained with uranyl acetate and lead citrate and viewed under an electron microscope Jeol Jem 1011 (Jem, Akishima, Tokyo, Japan) with an accelerating voltage of 80 kV.

### 2.12. Randomization and Blinding

Randomization was applied by randomly choosing the animals from their cages. Blinding was performed at different stages of the experiments: PTS or sham PTS procedure, sacrifice after a certain period post-(sham)-PTI, obtaining brain samples, microscopy, measurement, and statistical processing. Blinding was performed by different researchers.

### 2.13. Statistical Analysis

**Figure 1:**
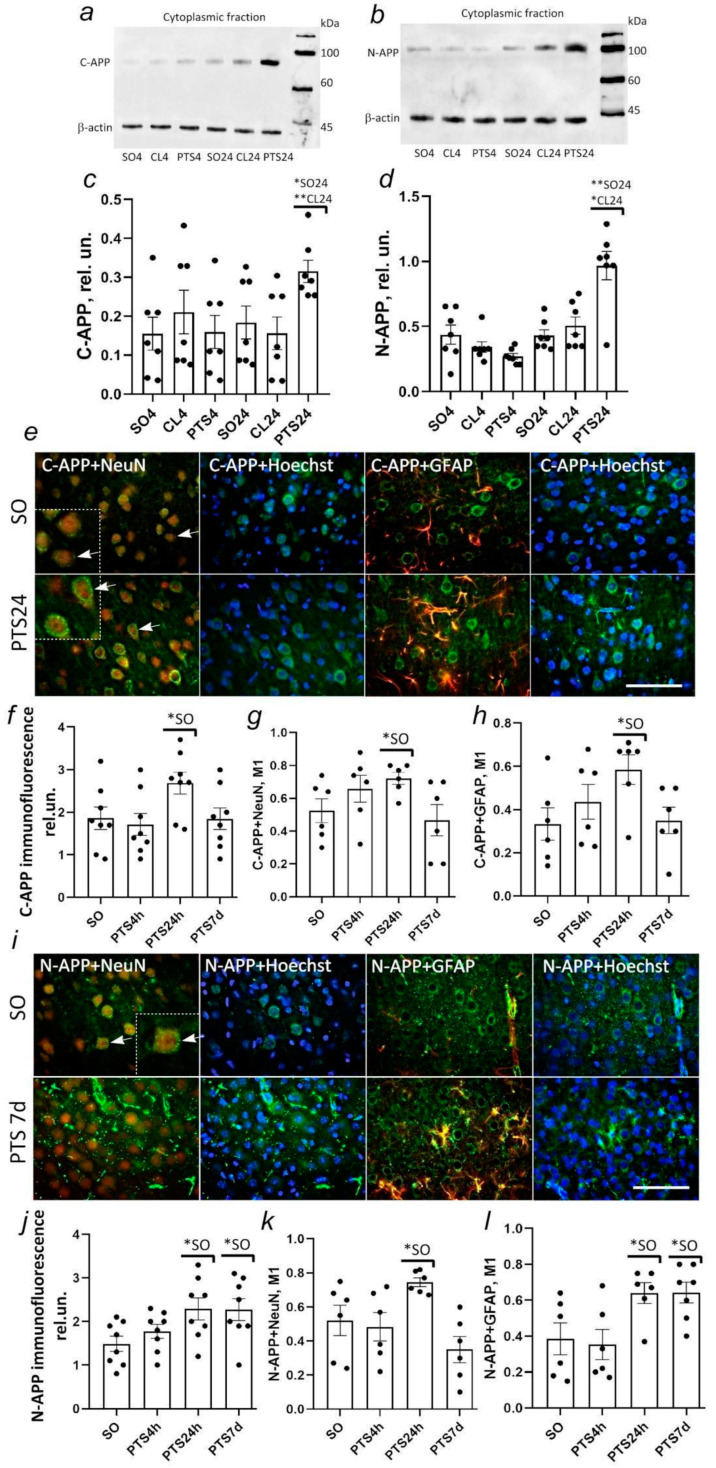
(**a**,**b**) Immunoblotting of C- and N-APP in the cytoplasmic fraction of the cortex of sham-operated animals (SO4 and SO24), at 4 (PTS 4 h) and 24 (PTS 24 h) hours after photothrombotic stroke in the cerebral cortex of rats and the contralateral cortex of the same rats (CL4 and CL24, respectively); (**c**,**d**) C- and N-APP level in relative units at different times after PTS; (**e**,**i**) Immunofluorescence of C- and N-APP (green); image overlay of C- and N-APP with NeuN (neuron marker, red), GFAP (astrocytes marker, red), and 33342 Hoechst (nuclei marker, blue). Scale bar 100 µm. Image overlay of ADAM10 with NeuN and with Hoechst. Scale bar 100 µm. Insert: enlarged image. Scale bar 50 µm. Arrows show granularity in the cytoplasm. (**f**,**j**) C- and N-APP fluorescence in penumbra cells after PTS and the cortex of sham-operated rats. (**g**,**k**) Localization of C- and N-APP in penumbra neurons. (**h**,**l**) Localization of C- and N-APP in penumbra astrocytes. The indices of the groups of sham-operated animals at 4 h, 24 h, and 7 days after irradiation had no statistically significant differences, therefore the indices of the three groups were combined (SO). One-way ANOVA. M ± SEM. *n* = 6–8. * *p* < 0.05; ** *p* < 0.01.

**Figure 2:**
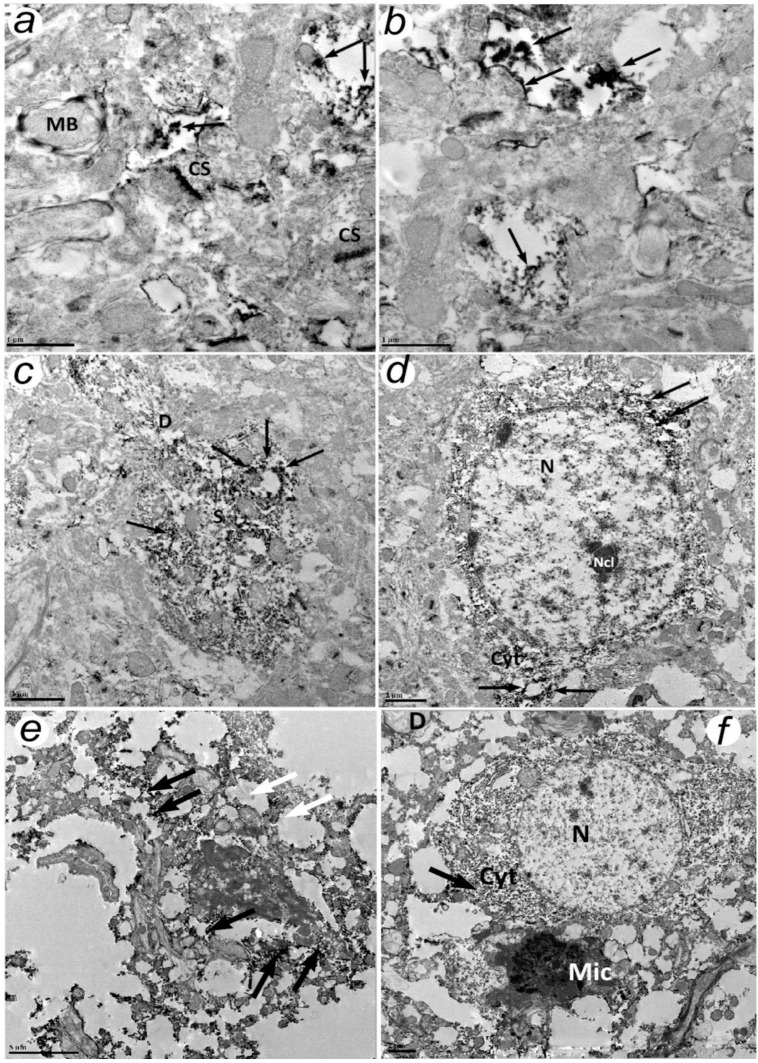
Electron immunohistochemistry with antibodies to C- and N-terminal fragments of APP protein in rat brain neocortex of the control group (**a**,**b**) and 4 h (**c**,**d**) or 24 h (**e**,**f**) after photothrombotic stroke. (**a**) Localization of APP (C-terminal domain): the medium amount of protein associated with cellular elements. (**b**) Localization of APP (N-terminal domain): filling of individual cell processes with APP. (**c**) APP (N-terminal domain) localization: numerous processes containing APP fragments. (**d**) APP (N-terminal domain) localization in neuronal cytoplasm. (**e**) The destruction of the neuropil, lysis of the cytoplasm of the cell and processes. (**f**) A neuron containing the APP and an APP-negative microglial cell. Legend: CS—chemical synapse, MB—myelin branch, S—neuronal soma, D—dendrite, N—nucleus, Ncl—nucleolus, Cyt—cytoplasm, Mic—microglia, the products of the reaction are indicated by arrows. Magnification: a and b ×10,000 (Scale bar 5 µm); c,d,f ×20,000 (Scale bar 2 µm); e ×50,000 (Scale bar 1 µm).

**Figure 3:**
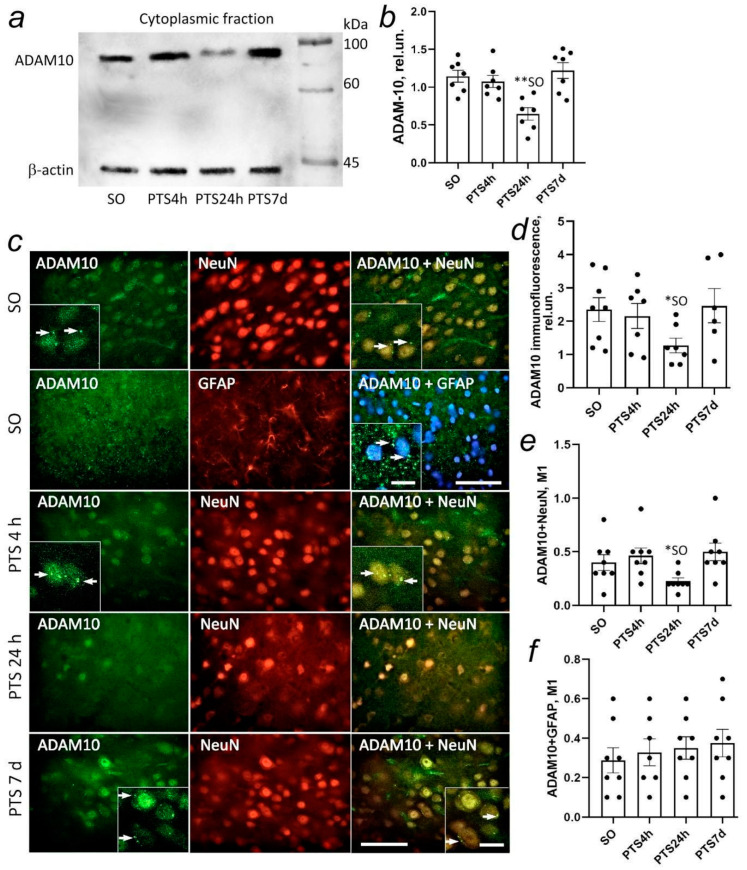
Changes in ADAM10 expression in the penumbra at 4 h, 24 h, and 7 days after PTS in the rat cerebral cortex (PTS 4 h, PTS 24 h, and PTS 7d, respectively) relative to the cortex of sham-operated rats (SO). (**a**) ADAM10 in the cytoplasmic fraction of the cortex of sham-operated animals (SO) and at 4 (PTS 4 h), 24 (PTS 24 h), and 7 days (PTS 7d) after PTS; (**b**) ADAM10 level in relative units at different times after PTS. The indices of the groups of sham-operated animals at 4 h, 24 h, and 7 days after irradiation had no statistically significant differences, therefore the indices of the three groups were combined. (**c**) Immunofluorescence and image overlay of ADAM10 (green), NeuN (red), and GFAP (red). Image overlay of ADAM10 with NeuN and with Hoechst. Scale bar 100 µm. Insert: enlarged image. Scale bar 50 µm. Arrows show granularity (clusters) in the area of the cytoplasmic membrane. (**d**) ADAM10 fluorescence in penumbra cells after PTS and in the cortex of sham-operated rats. (**e**) Localization of ADAM10 in penumbra neurons. (**f**) Localization of ADAM10 in penumbra astrocytes. One-way ANOVA. M ± SEM. *n* = 6–8. * *p* < 0.05 compared to the SO. ** *p* < 0.01 compared to the S.O.

**Figure 4:**
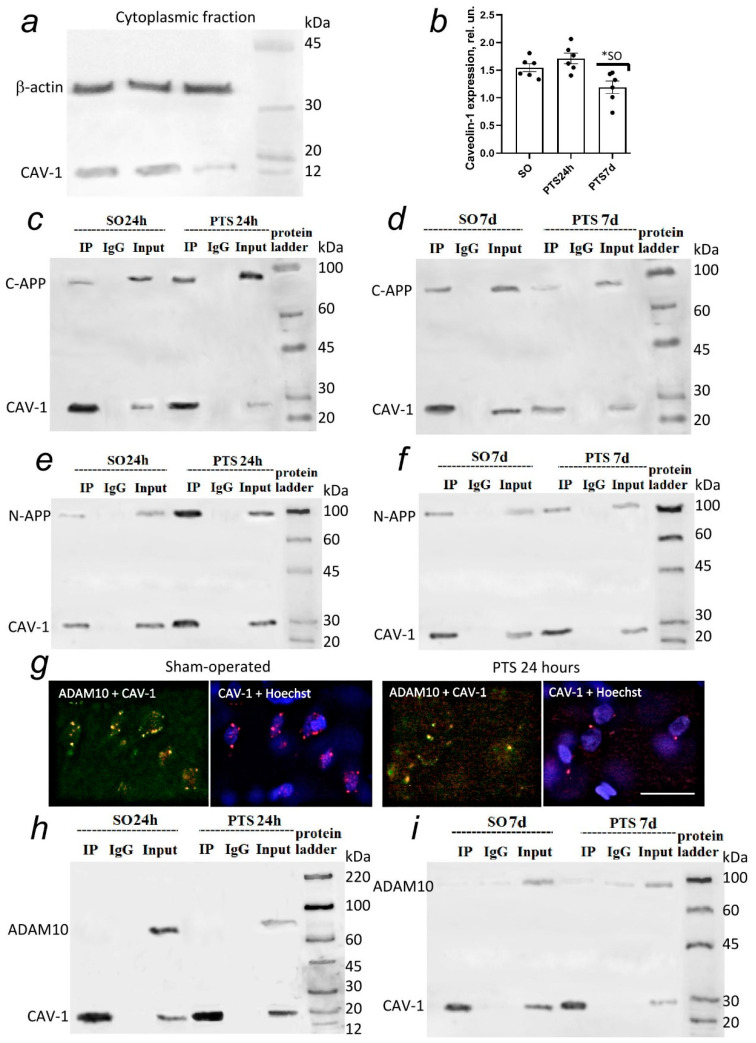
(**a**) Caveolin-1 (CAV-1) in the cytoplasmic fraction of the cortex of sham-operated animals (SO) and at 24 (PTS 24 h) and 7 days (PTS 7d) after PTS; (**b**) Caveolin-1 level in relative units at different times after PTS. The indices of the groups of sham-operated animals at 24 h, and 7 days after irradiation had no statistically significant differences. The result of Western blot analysis of co-immunoprecipitation of caveolin-1 protein and C-APP (**c**,**d**), or N-APP (**e**,**f**), or ADAM10 (**h**,**i**) in the cytoplasmic fraction of penumbra tissue at 24 h (PTS 24 h) and 7 days (PTS 7d) after PTS. Control group: cerebral cortex of sham-operated rats (SO24h or SO7d, respectively). Endogenous caveolin-1 proteins were immunoprecipitated (IP) with anti-caveolin-1 antibody, and co-precipitated C-APP or N-APP, or ADAM10 proteins were subsequently detected with anti-C-APP or N-APP, or ADAM10 antibodies, respectively. The expression level of caveolin-1 served as a Co-IP control. Negative control: normal IgG: 3 μg normal mouse IgG was added instead of anti-precipitating protein antibodies to exclude non-specific binding. Positive control: the original sample, not subjected to immunoprecipitation (input). To identify proteins on the blot, a molecular weight marker (protein ladder) was used. (**g**) Immunofluorescence and image overlay ADAM10 (green) and caveolin-1 (red) or caveolin-1 (red) and Hoechst (blue) in the cortex of sham-operated animals (SO) and at 24 h (PTS 24 h) after PTS. Scale bar 50 µm. One-way ANOVA. M ± SEM. *n* = 6–8. * *p* < 0.05 compared to the SO.

**Figure 5:**
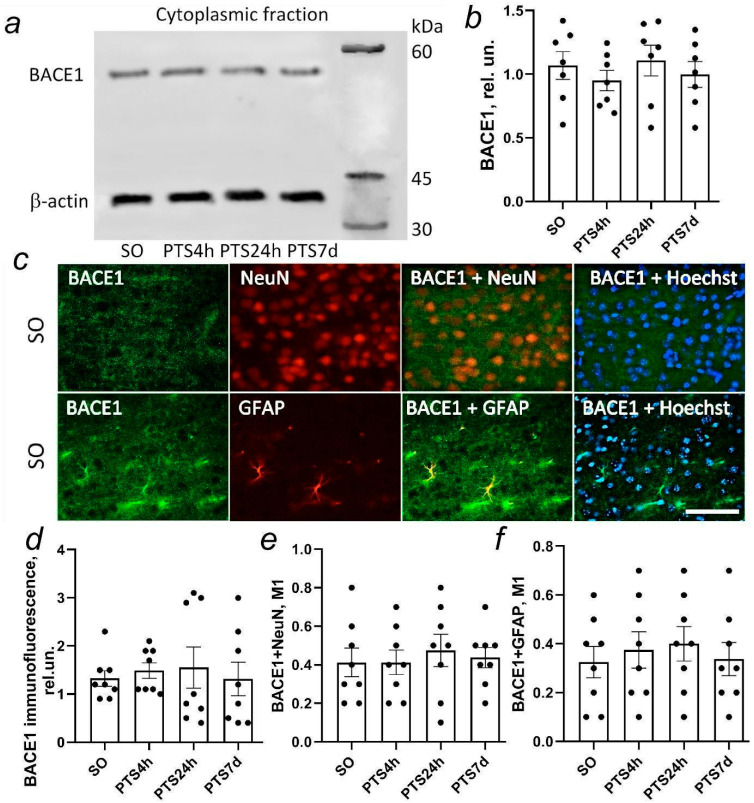
(**a**) Immunoblotting of BACE1 in the cytoplasmic fractions of the cortex of sham-operated animals (SO) and at four (PTS 4 h), 24 (PTS 24 h), and 7 days (PTS 7d) after PTS; (**b**) BACE1 level in relative units at different times after PTS. The indices of the groups of sham-operated animals at 4 h, 24 h, and 7 days after PTS had no statistically significant differences, therefore the indices of the three groups were combined. (**c**) Immunofluorescence of BACE1 (green), NeuN (neuron marker, red), and GFAP (astrocytes marker, red); image overlay of BACE1 with Neun, GFAP, and 33342 Hoechst (nuclei marker, blue). Scale bar 100 µm. (**d**) Changes (in relative units) in BACE1 fluorescence in penumbra cells after PTS and in the cortex of sham-operated rats. (**e**) Localization of BACE1 in penumbra neurons. (**f**) Localization of BACE1 in penumbra astrocytes. One-way ANOVA. M ± SEM. *n* = 7–8.

**Figure 6:**
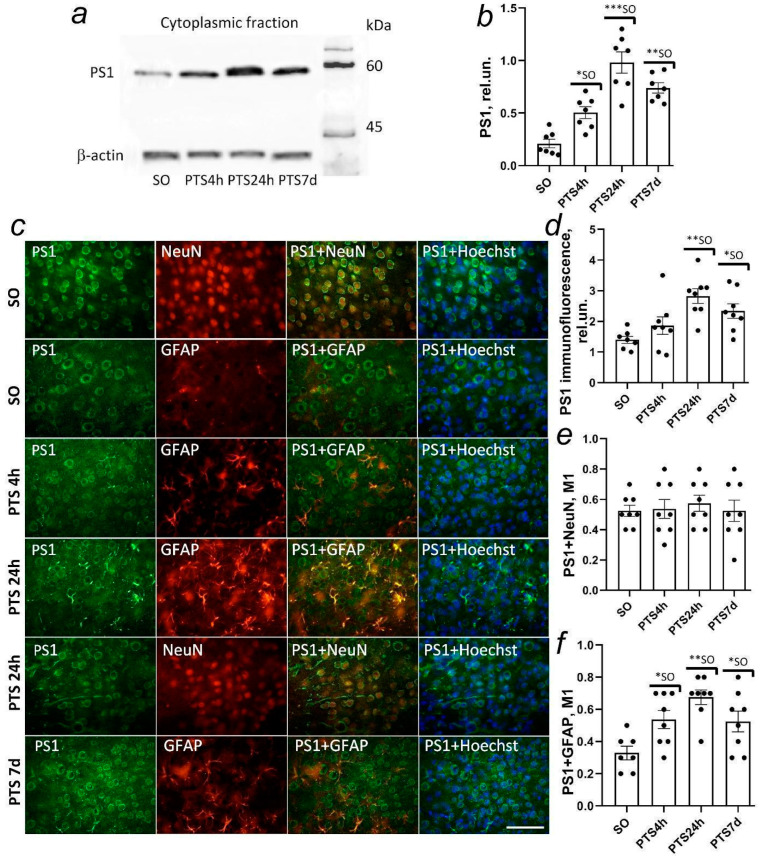
(**a**) Immunoblotting of presenilin-1 (PS1) in the cytoplasmic fraction of the cortex of sham-operated animals (SO) and at 4 h (PTS 4 h), 24 h (PTS 24 h), and 7 days (PTS 7d) after PTS; (**b**) PS1 level in relative units at different timepoints after PTS. The indices of the groups of sham-operated animals at 4 h, 24 h, and 7 days after PTS had no significant differences, therefore the indices of the three groups were combined. (**c**) Changes in PS1 expression in the penumbra at 4 h, 24 h, and 7 days after PTS in the rat cerebral cortex (PTS 4 h, PTS 24 h, and PTS 7 d, respectively) relative to the cortex of sham-operated animals (SO). Immunofluorescence of PS1 (green), GFAP (astrocytes marker, red), and NeuN (neuron marker, red); image overlay of PS1 with GFAP, Neun, and Hoechst 33342 (nuclei marker, blue). Scale bar 100 µm. (**d**) Changes (in relative units) in PS1 fluorescence in penumbra cells after PTS and in the cortex of sham-operated rats. (**e**) Localization of PS1 in penumbra astrocytes. (**f**) Localization of PS1 in penumbra neurons. One-way ANOVA. M ± SEM. *n* = 7–8. * *p* < 0.05 compared to the SO; ** *p* < 0.01 compared to the SO; *** *p* < 0.001 compared to the SO.

**Figure 7:**
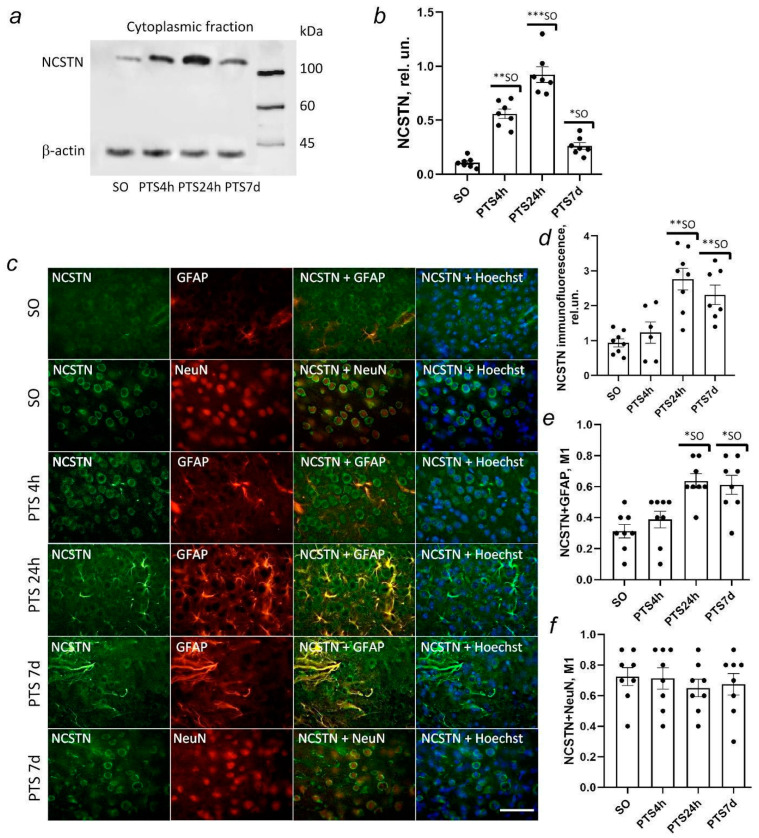
(**a**) Immunoblotting of nicastrin (NCSTN) in the cytoplasmic fraction of the cortex of sham-operated animals (SO) and at 4 h (PTS 4 h), 24 h (PTS 24 h), and 7 days (PTS 7d) after PTS; (**b**) Nicastrin level in relative units at different timepoints after PTS. The indices of the groups of sham-operated animals at 4 h, 24 h, and 7 days after PTS had no statistically significant differences, therefore the indices of the three groups were combined. (**c**) Immunofluorescence of nicastrin (NCSTN) (green), GFAP (astrocyte marker, red), and Neun (neuron marker, red); image overlay of NCSTN with Neun, GFAP, and Hoechst (nuclei marker, blue). Scale bar 100 µm. (**d**) Changes (in relative units) of nicastrin fluorescence in penumbra cells after PTS and in the cortex of sham-operated rats. (**e**) Localization of nicastrin in penumbra astrocytes. (**f**) Localization of NCSTN in penumbra neurons. One-way ANOVA. M ± SEM. n = 7–8. * *p* < 0.05 compared to the SO; ** *p* < 0.01 compared to the SO; *** *p* < 0.001 compared to the SO.

**Figure 8:**
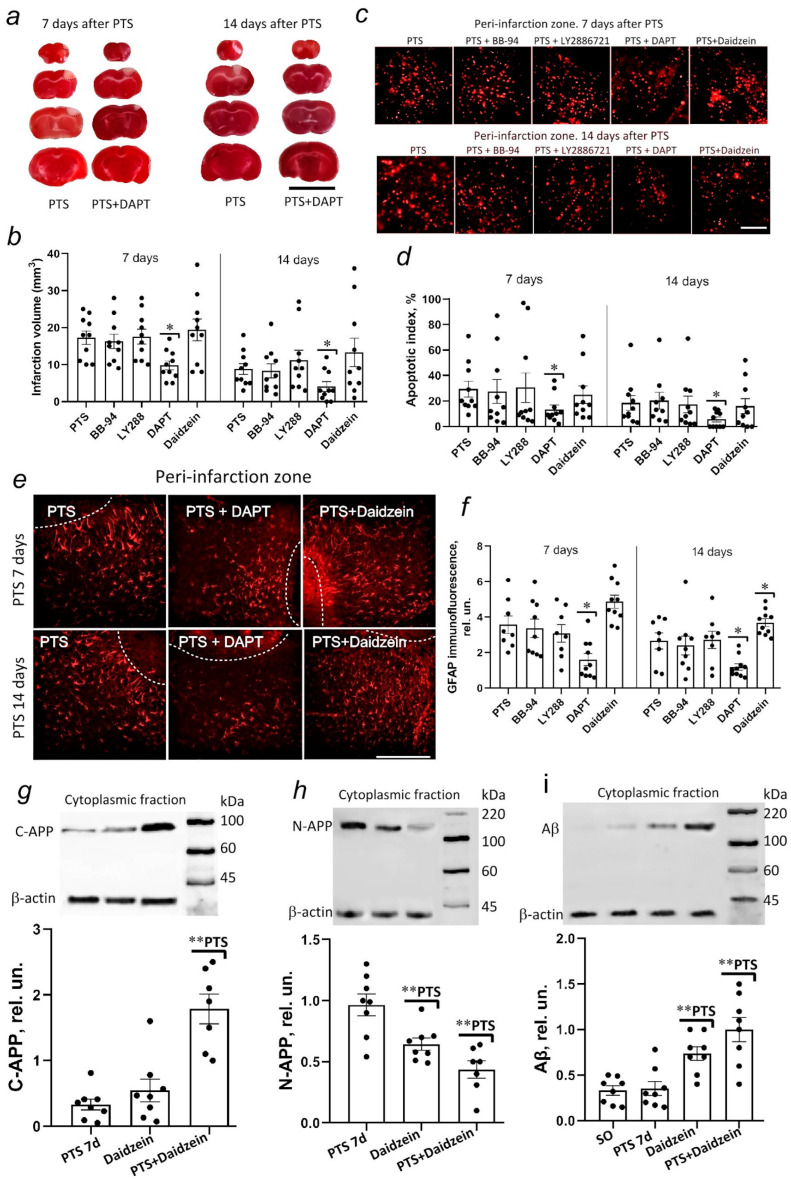
Effects of secretase inhibitors (LY2886721, an inhibitor of β-secretases BACE1 and BACE2; DAPT, an inhibitor of γ-secretase) and daidzein, an inhibitor of caveolin-1, on the volume of the infarction nucleus, apoptosis, and expression of GFAP in the peri-infarction region in the mouse cerebral cortex on days seven and 14 after PTS. (**a**) View of the mouse brain slices seven and 14 days after PTS and against the background of the DAPT administration, stained with TTS. The dotted line is the zone of infarction. (**b**) Average values of the infarct core volume (mm^3^) in the control groups (PTS without inhibitors) and the experimental groups (administration of inhibitors) seven and 14 days after PTS. Scale bar 1 cm. (**c**) Representative images of cortical areas stained with TUNEL (red fluorescence of apoptotic cells) seven and 14 days after PTS. Experimental groups: BB-94, an inhibitor of α-secretase ADAM10; LY2886721,an inhibitor of BACE1 and BACE2 β-secretases; DAPT is a γ-secretase inhibitor and daidzein, an inhibitor of caveolin-1. Scale bar 100 µm. (**d**) Changes in the apoptotic index (AI, %) in mice 7 and 14 days after PTS and after administration of inhibitors. (**e**) Immunofluorescence of GFAP (red) in the peri-infarction region of PTS in the mouse cerebral cortex on days 7 and 14 after PTS and against the background of the introduction of DAPT and daidzein. The dotted line is the zone of infarction. Scale bar 200 µm. (**f**) Changes (in relative units) of GFAP fluorescence in the peri-infarction region of PTS and against the background of the introduction of inhibitors. (**g**) Immunoblotting of C-APP in the cytoplasmic fraction of the cortex at 7 days (PTS 7d) after photothrombotic stroke in the cerebral cortex of mice and after administration of daidzein. (**h**) Immunoblotting of N-APP in the cytoplasmic fraction of the cortex at 7 days (PTS 7d) after photothrombotic stroke in the cerebral cortex of mice and after administration of daidzein. (**i**) Immunoblotting of amyloid beta (Aβ) in the cytoplasmic fraction of the cortex at 7 days (PTS 7d) after photothrombotic stroke in the cerebral cortex of mice and after administration of daidzein. T-test. M ± SEM. *n* = 7–10. * *p* < 0.05 compared to the PTS; ** *p* < 0.05 compared to the PTS.

In studies with a single effective factor (time post-PTS), differences between sample groups were statistically estimated using one-way ANOVA (Figure 1, Figure 2, Figure 3, Figure 4, Figure 5, Figure 6, Figure 7 and Figure 8). In studies where different-target inhibitors were used (secretase types in Figure 8), we applied the Student’s *t*-test.

For a posteriori (post-hoc) test in ANOVA, Holm–Sidak’s test was applied all-pairwise (for three 4 h groups and three 24 h independently in Figure 1c,d) or with the control comparison type (Figure 1, Figure 2, Figure 3, Figure 4, Figure 5, Figure 6, Figure 7 and Figure 8). Differences were considered significant at *p* < 0.05. Data were presented as mean ± SEM.

## 3. Results

### 3.1. APP Expression in the Rat Cerebral Cortex after PTS

According to the immunoblotting data, PTS stimulates the accumulation of the C-terminal APP fragment in the ischemic penumbra. In the cortex of control animals, the level of C-APP in the cytoplasmic fraction is not high (Figure 1a). At four hours after PTS, the level of C-APP in the penumbra tissue did not differ from the control. However, after 24 h, it significantly increased in the cytoplasmic fraction (Figure 1a,c). The level of N-APP was also low in the cytoplasmic fraction at 24 h but not at 4 h after PTS (Figure 1b,d).

The intensity of C-APP fluorescence increased by 45% (*p* < 0.05) at 24 h after PTS but it did not differ from the control level at 7 days after PTS (Figure 1f). An increase in the level of the C-terminal fragment of APP was observed both in neurons (Figure 1e,g) and in astrocytes at 24 h after PTS (Figure 1e,h). This was indicated by an increase in the co-localization coefficient of C-APP with the neuronal marker NeuN by 36% (Figure 1f) and with the astrocyte marker GFAP by 79% (Figure 1g).

According to immunofluorescent analysis, the increase in the level of N-APP at 24 h after PTS persisted for up to 7 days (Figure 1i,j). The level of the N-terminal fragment of APP was increased in neurons (by 44%) and in astrocytes (64%) at 24 h after PTS, and N-APP accumulated mainly in astrocytes on the 7th day after PTS (Figure 1i,l).

A more detailed picture of the intracellular distribution of the APP is provided by the electron immunohistochemistry data.

### 3.2. Subcellular Distribution of N- and C-Terminal Fragments of APP in Rat Brain Cells in Normal Conditions and on the First Day after Photothrombotic Stroke

In the brain samples of sham-operated rats after the immunohistochemical reaction with antibodies to APP fragments, it was shown that the reaction products in thin unmyelinated processes were visualized in the cerebral cortex. The N- and C-terminal fragments of APP were also found in large processes containing mitochondria. In addition to transected axons and dendrites along and across, there were active zones of chemical synapses in the field of view, while the reaction products seemed to accumulate in the postsynaptic part (Figure 2a).

In some cases, processes absorbed a larger amount of electron-dense flocculent material as a result of immunohistochemical reactions. The reaction products were also coupled to the plasma membranes of the processes (Figure 2c).

Electron microscopic examination of the brain neuropil 4 h after PTS and the study of the expression of the N- and C-ends of APP showed that both of them accumulate in the processes and the cytoplasm of individual neurons (Figure 2c).

The ubiquitous localization of APP fragments in the neuropil was observed at lower magnifications: in the longitudinally and transversely cut dendrites of neurons, as well as in processes with the absence of organelles and single mitochondria (presumably glial processes); in the zone of chemical synapses (Figure 2d).

Individual neurons containing APP fragments were observed. Such neurons contain nuclei without APP with several nucleoli and the cytoplasm with evenly distributed N- and C-terminal fragments of the APP (Figure 2c,d).

Significant tissue damage occurred 24 h after PTS: lysis of nuclei, destruction of cells and processes. At the same time, some elements of the destroyed tissue accumulated a significant amount of APP (Figure 2). Even in the destroyed tissue, the reaction products were localized mainly in the cytoplasm; neuropil elements, negative for APP, were also visualized (Figure 2f). For example, a neuron containing APP in the cytoplasm and a microglial cell with a dark nucleus negative for APP are localized among the destroyed tissue (Figure 2h). Thus, the reaction products of both C-terminal and N-terminal fragments of APP accumulate in thin processes, axons, and dendrites, and in chemical synapses. There were no differences in the localization or accumulation dynamics of the C- or N-terminal APP fragments at the ultrastructural level. At the level of individual neurons, APP fragments were associated with the cell cytoskeleton, rough endoplasmic reticulum, and ribosomes were absent in nuclei. The density of the reaction products depended on the time after exposure. Nerve cells contained the greatest amount of protein at 24 h after PTS. However, some glial cells and neurons did not accumulate APP. It is necessary to understand in further research what kind of cells do not contain APP and why.

### 3.3. ADAM10 Expression in the Rat Cerebral Cortex after Photothrombotic Stroke

According to the immunoblotting data on α-secretase, ADAM10 is mainly present in the cytoplasmic fraction of the penumbra tissue (Figure 3). A sufficiently high level of protein is noticed in sham-operated animals and at 4 h after PTS. The level of ADAM10 decreased 2-fold (*p* < 0.01) in the cytoplasmic fraction relative to sham-operated animals 24 h after PTS. PTS caused a significant increase in ADAM10 in the cytoplasmic fraction by 97% (*p* < 0.01) 7 day after PTS compared to the protein level at 1 day after PTS approaching the values of the group of sham-operated animals (Figure 3a,b).

A more detailed picture of the intracellular distribution of the protein is provided by immunofluorescence microscopy data. The immunofluorescence signal of ADAM10 in brain tissue is low. ADAM10 expression in neurons, but not in the penumbra astrocytes of sham-operated animals, is quite high (Figure 3c,d). The protein is localized mainly in the cytoplasm of neurons [34]. At 24 h after PTS, an almost 2-fold decrease in the level of ADAM10 compared with the control level is observed due to its decrease in neurons (Figure 5e) but not in penumbra astrocytes (Figure 5f). Noteworthy is the clustering of ADAM10 on the surface of the cytoplasmic membrane. It is known that APP, Aβ, BACE1, and presenilins were found in caveolae and the main structural component of caveolae, caveolin-1 (CAV-1), is able to interact directly with APP [25] and BACE1 [16,24]. At the same time, ADAM10 is largely excluded from lipid rafts [15,16] but APP processing by α-secretase was found in the caveolae of nerve cells [23]. Despite the contradictory data, there is no doubt that caveolae and caveolin-1 in nerve cells are directly involved in APP processing affecting the balance between amyloidogenic and non-amyloidogenic pathways. Thus, at the next stage of the study, we investigated the change in the level of caveolin-1 in penumbra cells at different time points after PTS and its co-localization with C- and N-terminal fragments of APP and ADAM10.

### 3.4. Caveolin-1 Expression and Its Interaction with C- and N-APP and ADAM10 in the Rat Cerebral Cortex after Photothrombotic Stroke

The level of caveolin-1 in the cytoplasmic fraction of penumbra cells decreased by 23% on the 7th day after PTS compared to sham-operated animals (Figure 4a,b). 

Only a small part of the C- and N-terminal APP forms a bond with caveolin-1 in the cytoplasmic fraction of the brain cortex cells of sham-operated animals (Figure 4c–f). Co-immunoprecipitation data have shown the increase in APP-caveolin-1 interaction at 24 h after PTS and its decrease on the 7th day after PTS that correlates with caveolin-1 decrease in penumbra cells at this time point (Figure 4c–f). Interestingly, co-immunoprecipitation occurred both in the case of using an antibody to C-APP that allows detection of the C-terminal residue of APP from amino acids 676 to 695 and in the case of using antibodies to the N-terminal residue of APP that allows the detection of fragments of sAPPα.

We also studied ADAM10 co-localization with caveolin-1 to describe the nature of ADAM10-containing clusters on the surface of the cerebral cortex cell membranes more accurately (Figure 3c). Co-localization of caveolin-1 with ADAM10 was observed by immunofluorescence microscopy (Figure 4g). However, the results of co-immunoprecipitation showed no direct interaction between caveolin-1 and ADAM10 after PTS (Figure 4h,j).

### 3.5. BACE1 Expression in the Rat Cerebral Cortex after Photothrombotic Stroke

β-secretase BACE1 is practically absent in the nuclear fraction of rat cerebral cortex cells. Our study of BACE1 expression by Western blot at different times after PTS did not reveal any change in BACE1 level in the cytoplasmic fractions of the penumbra tissue compared to the group of sham-operated animals (Figure 5a,b). 

BACE1 expression in neurons and astrocytes of the rat cerebral cortex was not high (Figure 5c,d), as indicated by Western blot data.

The results of the immunofluorescent analysis also did not reveal changes in BACE1 expression, either in neurons (Figure 5e) and astrocytes (Figure 5f) of the penumbra on the first day or in the early recovery period after a PTS (Figure 5d).

### 3.6. Presenilin-1 Expression in the Rat Cerebral Cortex after Photothrombotic Stroke

According to Western blot analysis, the level of presenilin-1 in both fractions of the cortex of sham-operated animals was low. There was an increase in the protein level in the cytoplasmic fraction of the penumbra tissue at 4 h after PTS (by 49%, *p* < 0.5) (Figure 6a,b). Protein overexpression in the cytoplasmic fraction increased 2.5-fold compared to the control (*p* < 0.01) at 24 h after PTS and 1.5-fold compared to the protein level at 4 h after PTS (*p* < 0.01) (Figure 6a,b). On day 7 after PTS, the protein level in the cytoplasm of penumbra cells significantly decreased compared to 24 h after PTS, returning to control values (Figure 6a,b).

Immunofluorescent analysis showed that the increase in presenilin-1 expression is associated with its increase in astrocytes but not in penumbra neurons (Figure 6c,e,f). The dynamics of changes in the co-localization coefficient of presenilin-1 with the astrocyte marker GFAP was almost the same as in the total cytoplasmic fraction (Figure 6b).

Presenilin-1 was localized exclusively in the cytoplasm in neurons both in normal conditions and after PTS (Figure 6c). An increase in the protein level on the first day after PTS persisted during the recovery period and was not associated with its growth in neurons since the co-localization of presenilin-1 with the neuronal marker NeuN did not differ significantly from that in sham-operated animals (Figure 6f).

### 3.7. Nicastrin Expression in the Rat Cerebral Cortex after Photothrombotic Stroke

Nicastrin belongs to the γ-secretase protein complex. Little is known about the function of nicastrin in neuronal injury after stroke. In the proteomic studies, we have shown that the expression of nicastrin in the penumbra increased on the first day after PTS [4]. 

The initial level of the nicastrin protein in the cytoplasmic fraction of the cortex of sham-operated animals was not high (Figure 7a). 

The protein level was practically not identified in the nuclear fraction. It increased 6-fold relative to sham-operated animals in the cytoplasmic fractions of the penumbra tissue at 4 h after PTS (Figure 7b). The protein level in the cytoplasmic fraction continued to rise and was 10-fold higher as compared to the control value (*p* < 0.01) 24 h after PTS and 6-fold higher as compared to the protein level 4 h after PTS (*p* < 0.01) (Figure 7a). The level of nicastrin significantly decreased at the 7th day after PTS compared to 4 and 24 h after PTS, but remained higher than the control group (*p* < 0.05) (Figure 7b).

The data from fluorescence microscopic studies confirm the conclusions of the Western blot analysis. Nicastrin is present in the cytoplasm of neurons and nuclei, cytoplasm, and astrocytes processes. Although there is a lot of protein in the brain cells of sham-operated animals, its level increased after PTS (Figure 7c). An increase in the level of nicastrin was observed at 24 h and persisted up to 7 days after PTS (Figure 7d). The increase in protein expression is associated with its high content in astrocytes (Figure 7e) but not in penumbra neurons (Figure 7f).

Thus, the expression of the main proteins of the γ-secretase complex, presenilin-1, and nicastrin, increased in penumbra astrocytes on the first day after PTS and in the early recovery period probably due to their involvement in the reaction of astrocytes to damage and during repair after PTS.

### 3.8. Effect of α-, β-, γ-Secretase and Caveolin-1 Inhibitors on Infarction Volume, Apoptosis Level, and Expression of GFAP in the Brain of Mice after Photothrombotic Stroke

Batimastat (BB-94) was used as an α-secretase inhibitor. The inhibitor molecule mimics the metalloproteinase substrate and therefore acts by competitive reversible inhibition [35]. BB-94 is used to study APP processing [36] and the role of its proteolytic products in memory and AD pathogenesis [37]. Metalloproteinases are currently considered effective antitumor agents in experiments with the administration of batimastat in rodent tumor models [35,38]. Batimastat is almost completely insoluble and therefore has very low oral bioavailability. Thus, the only way to administer batimastat is by direct injection into various body cavities (abdominal and pleural) [28]. In our work on PTS model in mice, batimastat (SML0041, Sigma-Aldrich) was dissolved in DMSO and administered to animals intraperitoneally at a dose of 50 mg/kg (or 3 mg/mL) one hour after irradiation once a day for five days [35,39,40,41]. It has been previously demonstrated that BB-94 efficiently enters the brain when administered intraperitoneally [41,42]. The selected dose of BB-94 has been shown to work in a mouse model of focal cerebral ischemia [31,33]. In addition, similar doses are effective in rodent tumor models [38]. However, we have not detected the effect of the drug on changes in infarct volume or the level of apoptosis of mouse cerebral cortex cells (Figure 8).

LY2886721 is a potent and selective active site inhibitor of β-secretase (BACE1,2) without inhibition of other proteases such as cathepsin D, pepsin, and renin [32,42]. Here, LY2886721 was dissolved in 6.7% DMSO and 5% Tween 20 in PBS and administered to animals intraperitoneally at a dose of 10 mg/kg/day for 5 days. As in the case of batimastat, we did not detect the effect of LY2886721 on changes in infarct volume or the level of apoptosis of mouse cerebral cortex cells after photothrombotic stroke (Figure 8).

DAPT (N-[N-(3,5-difluorophenacetyl)-1-alanyl]-Sphenylglycine t-butylester) is a γ-secretase inhibitor. The dose of DAPT was selected based on the literature data [43] and verified in preliminary experiments. The agent was dissolved in 5% DMSO and administered to animals intraperitoneally at a dose of 10 mg/kg/day for 5 days. Among the three secretase inhibitors studied, only DAPT reduced the infarction volume on the 7th and 14th days after PTS, preventing the growth of mouse cerebral cortex cell apoptosis in the area adjacent to the infarction zone (Figure 8).

In this work, we showed that photothrombotic stroke caused an increase in the level of γ-secretase protein subunits of presenilin-1 and nicastrin in astrocytes, but not in penumbra neurons. We evaluated reactive astrocyte marker GFAP expression in astrocytes of the perifocal region adjacent to the infarction zone after PTS and after the administration of the γ-secretase inhibitor DAPT. The inhibitor was shown to reduce the level of GFAP in cortical astrocytes (Figure 8g,h). At the same time, the effect was already seen on the 7th day after the administration of the agent and persisted up to 14 days after the PTS (Figure 8e,f).

Daidzein is known as an inhibitor of caveolin-1 [27,44]. Subcutaneous administration of daidzein from the 1st to 14th day after PTS had no significant effect on infarct volume or apoptosis of penumbra cells (Figure 8), but a decrease in caveolin-1 expression caused an increase in the level of GFAP in astrocytes of the perifocal region of infarction by 7 and 14 days after PTS (Figure 8e,f). The size of the cell body and the thickness of the astrocytic processes increased, the processes of astrocytes strongly overlapped, polarization towards the site of damage occurred, the position of each astrocyte was blurred, and a glial scar of considerable thickness was formed compared to the group that did not receive daidzein (Figure 8e). 

We also studied the effect of daidzein on C- and N-terminal APP and Aβ levels after PTS. Inhibitor administration slightly increased C-APP levels (+67%, *p* ≥ 0.05) in intact mice (Figure 8g). However, administration of daidzein at 7 days after PTS caused a 6-fold increase in the C-terminal fragment of APP that is known to be derived from the β-secretase processing of APP. A decrease in the expression of caveolin-1 together with the administration of daidzein caused an increase in the level of Aβ, especially after PTS by 233% (Figure 8j). According to immunofluorescent analysis, the level of the N-terminal fragment of APP that is mainly a product of α-secretase processing of APP increased significantly at 24 h after PTS and remained high for up to 7 days (Figure 1i,j). The administration of daidzein reduced the level of N-APP not only in intact mice (by 40%, *p* ≤ 0.05) but also by 60% after PTS (Figure 8h).

## 4. Discussion

**Figure 9:**
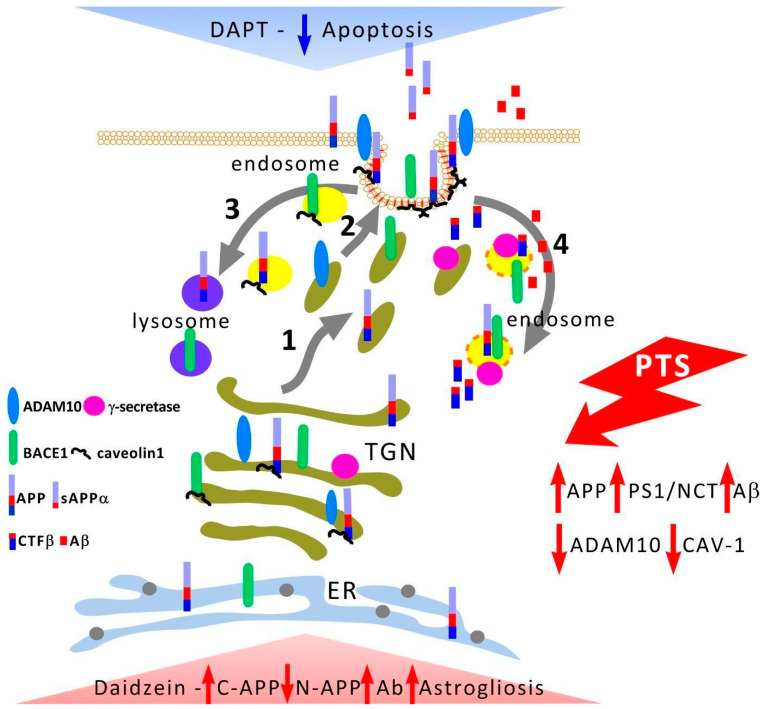
APP processing and effect of secretase and caveolin-1 inhibitors after PTS. Newly synthesized APP, BACE1, α-, and γ-secretases (1) are transported to the plasma membrane (2). Caveolae provide a platform for the regulation of APP processing. APP physically interacts with caveolin-1, providing its high concentration on the cytoplasmic membrane. APP is cleaved at the plasma membrane by α-secretase releasing sAPPα. After APP is cleaved by α-secretase, sAPPα is released into the extracellular space in a soluble form, while CTFα remains bound to the membrane where it is cleaved in lysosomes or further cleaved by γ-secretase. Most of APP, BACE1, and γ-secretase are internalized into early endosomes from the cell surface back into the cell and degraded in lysosomes (3). Delivery of APP or reduction of its internalization from the cell surface by binding to caveolin-1 promotes non-amyloidogenic APP processing. Amyloidogenic processing begins with the internalization of APP to endosomes. β-secretase, located on the endosomal membrane, cleaves APP at low pH into two forms in the lipid bilayer: the soluble fragment of APP (sAPPβ) and the membrane-bound β-carboxyl terminal fragment (CTFβ) (4). Further cleavage of CTFβ by γ-secretase produces Aβ monomers and the APP intracellular domain (AICD). The fate of APP along the amyloidogenic or non-amyloidogenic pathway largely depends on the co-localization of APP and secretases that can be influenced by caveolin-1, PTS causes an increase in the level of C- and N-terminal fragments of APP, proteins of the γ-secretase complex of presenilin-1 (PS1) and nicastrin (NCT) and Aβ with a decrease in the expression of ADAM10 and caveolin-1 (CAV-1). Decreased expression of caveolin1 by administering its inhibitor daidzein shifts APP processing towards Aβ synthesis that leads to hyperactivation of astrocytes. The administration of the γ-secretase inhibitor DAPT reduces the amount of PTS damage by reducing the level of apoptotic cell death in the peri-infarction area.

APP processing and traffic and secretases cleaving it are described in detail in some reviews [11,45,46]. After a ribosomal synthesis of the rough endoplasmic reticulum (RER), APP undergoes processing along the RER—Golgi apparatus (AG)—TGN (trans-Golgi network) vesicles—plasma membrane (PM) pathway (Figure 9). Only about 10% of APP reaches the neuronal membrane as part of TGN vesicles, while the rest is localized in AG and TGN. After incorporation into the PM, APP is internalized within a few minutes and enters endosomes. Part of its amount is recycled and again enters PM, while the other part is degraded in lysosomes. After passing through the TGN in neurons, transport vesicles are transported along the microtubules of axons and dendrites to the periphery of these neurites. It is important to know where APP proteolysis occurs and where its products are generated. A significant fraction of the sAPPα domain is generated by α-secretase inside TGN vesicles and it is exported to the external environment after their incorporation into the PM. BACE1 is first expressed in TGN vesicles and also is concentrated in lipid rafts of the plasma membrane. After reaching the PM, most of BACE1 is internalized and enters endosomes where the acidic environment promotes APP proteolysis (pH 4.5 is optimal for BACE1). Most of the vesicles carrying APP and BACE1 are spatially segregated, both in cultured neurons and in mouse or human brains [47]. Although both APP and BACE1 are synthesized via ER→Golgi, BACE1 is subsequently present in recycling endosomes (Figure 9). Thus, APP and BACE1 are transported in different vesicles and this simple spatial separation limits APP cleavage by BACE1 under normal conditions.

Here, we showed that C- and N-terminal fragments of APP are found not only in neurons but also in astrocytes after PTS. PTS caused the growth of both C- and N-terminal regions of APP already after 24 h. The level of the C-terminal fragment of APP decreased to control values by the 7th day after PTS and was localized in neurons, not astrocytes. A high level of N-APP (that could be predominantly a product of α-secretase (sAPPα) activity) persisted in astrocytes, but not in neurons at the 7th day after PTS. Interestingly, according to electron microscopy data, some neurons, astrocytes, and microglial cells did not contain APP. The reason for this selectivity remains to be seen. Little is known about the role of APP in astrocytes during cerebral ischemia. Although astrocytes express low levels of APP at rest, its level is strongly increased in models of brain injury where extensive gliosis occurs [48]. Astrocyte activation is an early sign of Alzheimer’s disease and may be a source of beta-amyloid that forms neuropathological plaques in Alzheimer’s disease [49]. Aβ is actively produced by reactive astrocytes as early as 3 days after MCAO, and peptide production decreases only 60 days after ischemia [50]. A 10 min cardiac arrest causes the growth of full-length amyloid precursor protein in reactive astrocytes up to 7 days after ischemia, and Aβ and C-terminus of APP only after 6 months when extensive loss of neurons and the onset of brain atrophy have been observed [51].

APP and its secretases’ distribution in different membrane domains and cell compartments (Figure 9) can affect the balance between amyloidogenic and non-amyloidogenic processing pathways. Cell line studies have shown that APP and BACE1 convergence occurs at the plasma membrane in detergent-resistant regions of the membrane [15,16] or possibly near it, but more recent data suggest that these two proteins converge within early endosomes [45,46,52]. Thus, the degree of co-localization of secretases remains unclear.

The proteinases of the “disintegrin and metalloproteinases” or ADAM family, such as ADAM9, ADAM10, TACE/ADAM17, and ADAM19, act as α-secretase in mammalian cells [14]. However, in neurons, α-secretase activity is associated with ADAM10 [53]. α-secretases cleave Notch receptors and ligands, tumor necrosis factor α, cadherins, the IL-6 receptor, EGF receptor ligands, and several other transmembrane proteins to release their extracellular domain [54]. Protein kinase C is known to promote the processing of α-secretases and the secretion of the APP ectodomain [11]. Cleavage of CTFα by γ-secretase releases the 3 kD p3 peptide and AICD. Studies have shown that p3 can have neurotoxic effects such as neuronal apoptosis [55]. Chronic cerebral hypoperfusion caused an increase in the levels of sAPPα, ADAM10, and ADAM17 in the hippocampus of rats against the background of an even more significant increase in the levels of sAPPβ, BACE, and BACE1 contributed to the promotion of the amyloidogenic pathway of APP processing and caused cognitive impairment [34]. It is possible that the decrease in ADAM10 expression that we observed after PTS is associated with a general decrease in protein biosynthesis needed during the most acute period of stroke. A comparison of the behavior of adult mice showed that the loss of ADAM10 in A10cKO mice leads to a decrease in neuromotor abilities and a decrease in learning ability that was associated with a change in the activity of neurons in the CA1 region of the hippocampus and impaired synaptic function [56]. Histological and ultrastructural analysis of the brain of A10cKO mice revealed astrogliosis, microglial activation, and disturbances in the number and morphology of postsynaptic structures [56]. In a rat PTS model, a decrease in the enzyme in neurons occurred on the first day after PTS but by the seventh day, the protein level was restored to control values. At the same time, ADAM10 expression decreased in neurons, but not in astrocytes, where N-APP also remained high (Figure 1i,l). Probably, the increase in immunofluorescence of N-APP both in neurons on the first day after PTS and in astrocytes on the 7th day after PTS was not associated with the accumulation of ADAM10 activity products sAPPα, but was associated either with the accumulation of a full-length protein APP or accumulation of products of further processing of the amino-terminal region of APP that could be nonspecifically detected by the antibody we used. 

Inhibition of BACE1 cleavage of neuregulin-1 (NRG1) and possibly neuregulin-3 (NRG3) causes a decrease in the thickness of the myelin sheath of axons of both peripheral nerves (sciatic nerve) and central optic nerves and impairs remyelination of injured nerves [57]. Proteolytic processing of the neuregulin-1 (NRG1) BACE1 protein is associated with the activation of ErbB receptor tyrosine kinases. This signaling pathway is involved in synapse formation, plasticity, neuronal migration, myelination of central and peripheral axons, and in the regulation of neurotransmitter expression and function. It is possible that seven days after the neurotrauma time point was an insufficient time to activate the expression of the enzyme since the available data indicate that high BACE1 activity in neurons may be associated with repair processes after brain cell damage.

γ-secretase catalyzes the final cleavage of APP with AICD and either Aβ in the case of the amyloidogenic pathway of APP proteolysis or p3 in the non-amyloidogenic variant of APP proteolytic formation. γ-secretase is composed of four integral membrane proteins: presenilin (PS) 1 or 2, nicastrin (NCSTN), PEN-2, and APH-1. Assembly of the complex begins with the stabilization of PS with nicastrin and APH-1, after which, the last component of the PEN-2 protein complex is added [58]. Biochemical studies show that PS1 and PS2 (or APH-1a and APH-1b and their alternatively spliced forms) never coexist in the same complex, suggesting that there are at least six different γ-secretase complexes in humans. PS1 is located mainly in the endoplasmic reticulum [59]. However, significant amounts of PS1 bound to NCSTN were found in the plasma membrane and endosomes/lysosomes, indicating that completely assembled complexes leave the endoplasmic reticulum and translocate to the plasma membrane. All four components of the γ-secretase complex are localized in the active form on the plasma membrane and lysosomes [59]. Our studies have shown that the expression of proteins of the γ-secretase complex, PS1 and nicastrin, increases in astrocytes, but not in penumbra neurons on the first day after PTS and remains high up to seven days after PTS. However, in the model of global ischemia, PS1 expression decreased from two up to seven days but the trend was reversed on day 30 [60]. 

Electron microscopy shows that the C- and N-terminal fragments of APP were associated with the plasma membranes of the processes of nerve cells. Our attention was drawn to the pronounced clustering of APP and ADAM10 but not BACE1. This is unexpected since BACE1 and presenilin1 (PS1), the catalytic unit of γ-secretase, are localized mainly in detergent resistant membranes (DRM) or lipid rafts, while ADAM10 is localized mainly in non-lipid raft domains.

Caveolae are a subset of lipid rafts that are characterized by small membrane invaginations and the presence of caveolin-1 [23,25,26,27]. Caveol-like membrane domains have been characterized in nerve cells [23,61,62]. In Alzheimer’s disease, caveolar dysfunction can cause a decrease in α-secretase activity and accumulation of toxic amyloid Aβ peptide [15,25]. Caveolin-1 is known to physically interact with APP [23,25] and BACE1 [24], and overexpression of caveolin-1 attenuated γ-secretase-mediated proteolysis of APP and Notch [63]. Caveolin-1 was weakly expressed in rat brain cells, and PTS caused a further decrease in its level. Immunofluorescent analysis indicates a high co-localization of caveolin-1 with ADAM10 after PTS. However, the results of immunoprecipitation indicate an increase in the interaction between caveolin-1 and C-APP and, to a much lesser extent, between N-APP, as well as the absence of direct physical interaction between caveolin-1 and ADAM10 (Figure 4). Our results confirm the data that the caveolin-1 binding motif is located on the C-terminal cytoplasmic tail of APP [23]. The observed co-localization in fragments of APP and ADAM10 with caveolin-1 indicates the localization of APP and ADAM10 in areas of brain cells rich in caveolin both on the membrane and outside it. Our data indicate the accumulation of APP fragments in axons and dendrites and the zones of chemical synapses. It is assumed that axonal APP is concentrated in the caveolar structures of neurons [64]. Caveolin-1 can act independently of caveolae in ischemia [65]. Caveolin can be found in the trans-Golgi network (TGN) in the cytosol or separate structures, such as caveosomes (early endosomes) and TGN, which can be the site of APP processing to form Aβ [66]. Thus, the balance of caveolin-1 during ischemia may affect APP processing and the degree of damage to brain cells after ischemia. Caveolin-1 may play an important role in protecting the brain from stroke. Mice with caveolin-1 knockout had less lesions, lower neurological deficits, and less cerebral edema after intracerebral hemorrhage [67] but caveolin-1 knockout mice showed a high level of apoptotic death of penumbra cells after ischemic stroke [68].

We carried out an inhibitory analysis to understand the significance of the detected changes in the expression of α-, β-, γ-secretase proteins, and caveolin-1.

Batimastat, or BB-94, was used as an α-secretase inhibitor. Some studies have demonstrated the neuroprotective effect of the inhibitor with a decrease in infarct volume [31], an improvement in neurological functions, and a decrease in mortality in various models of ischemic stroke in rats and mice [69,70], as well as excitotoxic damage to neurons in cell culture [71]. However, we have not detected the effect of the drug on changes in infarction volume or the level of apoptosis in cells of the mouse cerebral cortex.

LY2886721 demonstrates an effective dose-dependent decrease in the level of Aβ and sAPPβ in different experimental models: in HEK293 cells with the APP751 mutation; in primary cortical neurons of PDAPP-mutated mice [72]; in vivo animal models (3–30 mg/kg PDAPP mice, 1.5 mg/kg beagle dogs; orally) [22,52,53,54]. In transgenic mice, doses of 3–30 mg/kg reduced Aβ levels by 20–65%. The effect lasted up to nine hours after the application of the drug. A decrease in amyloid production has been observed in plasma and cerebrospinal fluid after the administration of LY2886721 [32,42]. In a beagle dog model, oral administration (1.5 mg/kg) showed a significant and persistent reduction in Aβ levels in the cerebrospinal fluid [73]. However, we could not detect the effect of LY2886721 on changes in infarct volume or the level of apoptosis of mouse cerebral cortex cells after ischemic stroke.

DAPT (N-[N-(3,5-difluorophenacetyl)-1-alanyl]-Sphenylglycine t-butylester) is a γ-secretase inhibitor. Among the three secretase inhibitors studied, only DAPT reduced the infarct volume on days 7 and 14 after PTS, preventing the increase in apoptosis of mouse cerebral cortex cells in the area adjacent to the infarction zone. This inhibitor was used to treat neurodegenerative diseases and modulated the differentiation of progenitor neurons and apoptotic cascades in neurons during cerebral ischemia [33]. DAPT protects the brain from cerebral ischemia [74] by influencing inflammatory processes, suppressing the expression of NF-κB, a family of transcription factors involved in ischemic injury, promoting inflammatory processes and inducing neuronal apoptosis [75,76,77]. DAPT has been shown to have an expressed neuroprotective effect in a mouse model of ischemia/reperfusion (I/R) caused by occlusion of the middle cerebral artery. DAPT significantly improved neurobehavioral performance and reduced neuronal morphological damage. It reduced the level of GFAP as well as the number of apoptotic cells by reducing the content of interleukin-6 and tumor necrosis factor-α [77]. Here, we also showed that photothrombotic stroke causes an increase in the level of γ-secretase proteins PS1 and nicastrin mainly in astrocytes and the administration of the inhibitor reduces the level of GFAP. It is most likely that inhibition of γ-secretase enhances the anti-inflammatory response and reduces the activation of astrocytes, contributing to the decrease in the level of apoptosis and, as a consequence, the amount of damage after ischemia. A possible mechanism for this drug effect could be a decrease in the synthesis of Aβ that can activate apoptosis both externally and internally [78]. A decrease in the level of Aβ in astrocytes that increases after ischemia [50,51] can also contribute to the reduction of astrogliosis after PTS against the background of DAPT administration.

Caveolin-1-deficient cells are known to exhibit significantly increased co-localization of γ-secretase with clathrin-coated non-caveolar endocytic vesicles [63] and a redistribution of γ-secretase between caveolar and non-caveolar membranes may stimulate Aβ formation against the background of a decrease in caveolin-1 levels that is observed at the 7th day after PTS. The administration of daidzein caused an increase in the level of C-APP and Aβ and a decrease in the N-terminal fragment after PTS (Figure 8g,h,j). Thus, the present study shows that APP is concentrated in caveolae-rich membrane regions not only in the cytoplasm but also may be in endosome membranes (caveosomes) and in TGN where caveolin-1 ensures the concentration of APP in these membrane microdomains increasing the activity of α-secretase. It remains to be determined whether caveolin-1 is part of the retromer complex or interacts with it to sort out APP from β- and γ-secretase [79] in late endosomal compartments, resulting in decreased Aβ production. Moreover, the inactivation of γ-secretase will help to reduce the formation of Aβ peptides. However, the transport of caveolin-1 is also regulated by presenilins [80]. PS1 deficiency can lead to a serious loss of caveolae slowing repair processes after ischemia and activating astrocytes that make blocking of the expression of presenilin-1 (rather than a decrease in γ- secretase activity) unacceptable. We showed that the decrease in the level of caveolin-1 that is caused by the administration of daidzein contributed to the activation of astrocytes and the development of astrogliosis in the long term. A study of Cav-1 knockout mice showed reduced neovascularization and modified astrogliosis without proper glial scar formation around the infarct core 3 days after stroke [81]. In addition, knockout or knockdown of caveolin-1 increased blood–brain barrier (BBB) permeability and cell damage after cerebral ischemia-reperfusion by activating the NO/Cav-1/MMP signaling cascade [82]. 

The shift of APP processing towards the amyloidogenic pathway and the formation of Aβ, as well as full-length APP, can be a link between cardiovascular and neurodegenerative diseases [83,84]. The accumulation of full-sized APP in the mitochondria of brain cells, as well as cells of peripheral tissues, causes mitochondrial dysfunction and impairs energy metabolism [83]. APP is involved in the activation of endothelial cells and increases the expression of pro-inflammatory proteins, cyclooxygenase-2, and vascular cell adhesion molecule-1, as well as the cytokine IL-1β [85]. The accumulation of Aβ in the blood, vascular walls, and heart causes endothelial activation, inflammation and tissue damage, and impaired glucose metabolism, which contributes to the development of atherosclerosis and the formation of blood clots, chronic inflammation, and diabetes mellitus [83,84]. APP and its processing products are the link between aging and cardiovascular disease, and possibly vice versa. On the one hand, a violation of the blood–brain barrier during a stroke can cause an increase in APP and Aβ in the blood and their accumulation in the tissues of peripheral organs, disrupting metabolism. On the other hand, an increase in the level of Aβ in the brain after a stroke and its toxicity to endotheliocytes causes the development of inflammation, disruption of endothelial repair processes, cytokine-induced damage to BBB component cells, alteration of leukocyte–endothelial interactions, and the development of neurodegeneration. In this regard, in the future it will be interesting to study the balance of markers of BBB permeability impairment (neuron-specific enolase (NSE), GFAP, α-glycoprotein, etc.), inflammation markers in the peripheral blood after a stroke and their correlation with the level of APP and Aβ in the brain and blood in the recovery period.

Thus, inhibitory analysis showed that the decrease in ADAM10 expression in neurons and the increase in the expression of PS1 γ-secretase complex nicastrin against the background of the decrease in caveolin-1 in astrocytes promote a shift in APP processing towards the amyloidogenic pathway, which would lead to neuronal death and the development of astrogliosis and inflammation in the early recovery period after PTS. DAPT may be considered as a potential drug for stroke treatment. However, DAPT and LY2886721 inhibited the γ-secretase complex containing PS1 rather than the γ-secretase complex with PS2 in humans (in contrast to the results obtained in mice) [86]. The potential side effects caused by blocking Notch signaling must be considered [87]. 

## 5. Conclusions 

The processing of APP is involved in the pathogenesis of many neurodegenerative disorders. We investigated the expression of APP and its processing proteases such as α-secretase of ADAM10, β-secretase of BACE1, γ-secretase subunits of PS1 and NCT, and caveolin-1 in a PTS model. The results showed an increase in the level of N- and C-terminal fragments of APP in the cytoplasm of ischemic penumbra cells 24 h after PTS and their co-immunoprecipitation with caveolin-1. α-secretase of ADAM10 was proven to be mainly present in the cytoplasmic fraction of the penumbral tissue and was increased significantly in neurons at 24 h after PTS. However, no significant difference was found at different times after PTS for the expression of β-secretase of BACE1, although the expression of BACE1 in neurons and astrocytes of the rat cerebral cortex seemed relatively low. Interestingly, both γ-secretase subunits of PS1 and NCT were shown to increase in astrocytes at 24 h after PTS. The caveolin-1 inhibitor daidzein shifted APP processing towards Aβ synthesis, which caused astroglial activation. To further discover the roles of these secretases, Batimastat (BB-94), an α-secretase inhibitor, LY2886721, a β-secretase inhibitor, and DAPT, a γ-secretase inhibitor, were used in a PTS mice model. Among these, only DAPT can reduce the infarct volume on days 7 and 14 after PTS, preventing the growth of mouse cerebral cortex cell apoptosis in the area adjacent to the infarction zone. Regarding the positive correlation of the expression of APP, α-secretase of ADAM10, and γ-secretase subunits of PS1 and NCT after PTS, further research to reveal the exact roles of these proteins in stroke would be interesting and valuable to explore.

In conclusion, we demonstrated that a photothrombotic stroke led to reduced expression of ADAM10 α-secretase in neurons and increased the levels of the γ-secretase subunits of PS1 and NCT in astrocytes. Furthermore, the inhibitory assay showed that only the γ-secretase inhibitor of DAPT reduces GFAP levels and decreases brain infarct volume, suggesting γ-secretase appears to be a therapeutic target and its inhibitor of DAPT may have the therapeutic potential for the treatment of stroke. An important direction will be the development of very selective γ-secretase modulators targeting one subunit of the enzyme.

## Data Availability

Data are available on demand to corresponding author S.V. Demyanenko; B. He.

## Supplementary Materials

The following supporting information can be downloaded at: https://www.mdpi.com/article/10.3390/biomedicines10102655/s1, Figure S1. An example of Western blot evaluation of the purity of the obtained nuclear and cytoplasmic fractions of the cerebral cortex of sham-operated rats, penumbra tissue after 4 (PTS 4 h), 24 (PTS 24 h) hours, and 7 days (PTS 7d) after photothrombotic stroke (PTS) in rats. The acetylated histone H4 protein (ac-H4) was used as a nuclear fraction marker. Anti-acetyl-Histone H4 produced in rabbit (#06-866, Merck) diluted 1:500 was used. The protein glyceraldehyde-3-phosphate dehydrogenase (GAPDH) was used as a marker of the cytoplasmic fraction. Anti-GAPDH antibody produced in rabbit (G9545, Sigma-Aldrich) diluted 1:1000 was used. Table S2. List of primary antibodies used in this study. Primary antibodies used for WB assay are listed.

## References

[1] Orellana-Urzúa S., Rojas I., Líbano L., Rodrigo R. (2020). Pathophysiology of Ischemic Stroke: Role of Oxidative Stress. Curr. Pharm. Des..

[2] Iadecola C., Anrather J. (2011). Stroke research at a crossroad: Asking the brain for directions. Nat. Neurosci..

[3] Hankey G.J. (2017). Stroke. Lancet.

[4] Demyanenko S., Uzdensky A. (2017). Profiling of Signaling Proteins in Penumbra After Focal Photothrombotic Infarct in the Rat Brain Cortex. Mol. Neurobiol..

[5] Jacobsen K.T., Iverfeldt K. (2009). Amyloid precursor protein and its homologues: A family of proteolysis-dependent receptors. Cell. Mol. Life Sci..

[6] Guo Q., Wang Z., Li H., Wiese M., Zheng H. (2012). APP physiological and pathophysiological functions: Insights from animal models. Cell Res..

[7] Dawkins E., Small D.H. (2014). Insights into the physiological function of the β-amyloid precursor protein: Beyond Alzheimer’s disease. J. Neurochem..

[8] Müller U.C., Deller T., Korte M. (2017). Not just amyloid: Physiological functions of the amyloid precursor protein family. Nat. Rev. Neurosci..

[9] Hefter D., Draguhn A. (2017). APP as a Protective Factor in Acute Neuronal Insults. Front. Mol. Neurosci..

[10] Pluta R., Ułamek-Kozioł M., Januszewski S., Czuczwar S.J. (2020). Participation of Amyloid and Tau Protein in Neuronal Death and Neurodegeneration after Brain Ischemia. Int. J. Mol. Sci..

[11] Haass C., Kaether C., Thinakaran G., Sisodia S. (2012). Trafficking and Proteolytic Processing of APP. Cold Spring Harb. Perspect. Med..

[12] Vincent B., Govitrapong P. (2011). Activation of the α-secretase processing of AβPP as a therapeutic approach in Alzheimer’s disease. J. Alzheimer’s Dis..

[13] Vincent B. (2016). Regulation of the α-secretase ADAM10 at transcriptional, translational and post-translational levels. Brain Res. Bull..

[14] Endres K., Deller T. (2017). Regulation of Alpha-Secretase ADAM10 In vitro and In vivo: Genetic, Epigenetic, and Protein-Based Mechanisms. Front. Mol. Neurosci..

[15] Kojro E., Gimpl G., Lammich S., Marz W., Fahrenholz F. (2001). Low cholesterol stimulates the nonamyloidogenic pathway by its effect on the alpha -secretase ADAM 10. Proc. Natl. Acad. Sci. USA.

[16] Harris B., Pereira I., Parkin E. (2009). Targeting ADAM10 to lipid rafts in neuroblastoma SH-SY5Y cells impairs amyloidogenic processing of the amyloid precursor protein. Brain Res..

[17] Carey R.M., Blusztajn J.K., Slack B.E. (2011). Surface expression and limited proteolysis of ADAM10 are increased by a dominant negative inhibitor of dynamin. BMC Cell Biol..

[18] Zhang X., Zhou K., Wang R., Cui J., Lipton S.A., Liao F.-F., Xu H., Zhang Y.-W. (2007). Hypoxia-inducible factor 1alpha (HIF-1alpha)-mediated hypoxia increases BACE1 expression and beta-amyloid generation. J. Biol. Chem..

[19] Guglielmotto M., Aragno M., Autelli R., Giliberto L., Novo E., Colombatto S., Danni O., Parola M., Smith M.A., Perry G. (2009). The up-regulation of BACE1 mediated by hypoxia and ischemic injury: Role of oxidative stress and HIF1alpha. J. Neurochem..

[20] Carroll C.M., Li Y.M. (2016). Physiological and pathological roles of the γ-secretase complex. Brain Res. Bull..

[21] Lee J., Retamal C., Cuitiño L., Caruano-Yzermans A., Shin J.E., van Kerkhof P., Marzolo M.P., Bu G. (2008). Adaptor protein sorting nexin 17 regulates amyloid precursor protein trafficking and processing in the early endosomes. J. Biol. Chem..

[22] Mañucat-Tan N.B., Saadipour K., Wang Y.J., Bobrovskaya L., Zhou X.F. (2019). Cellular Trafficking of Amyloid Precursor Protein in Amyloidogenesis Physiological and Pathological Significance. Mol. Neurobiol..

[23] Ikezu T., Trapp B.D., Song K.S., Schlegel A., Lisanti M.P., Okamoto T. (1998). Caveolae, plasma membrane microdomains for alpha-secretase-mediated processing of the amyloid precursor protein. J. Biol. Chem..

[24] Hattori C., Asai M., Onishi H., Sasagawa N., Hashimoto Y., Saido T.C., Maruyama K., Mizutani S., Ishiura S. (2006). BACE1 interacts with lipid raft proteins. J. Neurosci. Res..

[25] Head B.P., Peart J.N., Panneerselvam M., Yokoyama T., Pearn M.L., Niesman I.R., Bonds J.A., Schilling J.M., Miyanohara A., Headrick J. (2010). Loss of caveolin-1 accelerates neurodegeneration and aging. PLoS ONE.

[26] Tang W., Li Y., Li Y., Wang Q. (2021). Caveolin-1, a novel player in cognitive decline. Neurosci. Biobehav. Rev..

[27] Gupta A., Sharma A., Kumar A., Goyal R. (2019). Alteration in memory cognition due to activation of caveolin-1 and oxidative damage in a model of dementia of Alzheimer’s type. Indian J. Pharmacol..

[28] Uzdensky A.B. (2018). Photothrombotic Stroke as a Model of Ischemic Stroke. Transl. Stroke Res..

[29] Demyanenko S.V., Panchenko S.N., Uzdensky A.B. (2015). Expression of Neuronal and Signaling Proteins in Penumbra around a Photothrombotic Infarction Core in Rat Cerebral Cortex. Biochem..

[30] Uzdensky A., Demyanenko S., Fedorenko G., Lapteva T. (2017). Photothrombotic infarct in the rat brain cortex: Protein profile and morphological changes in penumbra. Mol. Neurobiol..

[31] Asahi M., Asahi K., Jung J.-C., Del Zoppo G.J., Fini M.E., Lo E.H. (2000). Role for matrix metalloproteinase 9 after focal cerebral ischemia: Effects of gene knockout and enzyme inhibition with BB-94. J. Cereb. Blood Flow Metab..

[32] May P.C., Willis B.A., Lowe S.L., Dean R.A., Monk S.A., Cocke P.J., Audia J.E., Boggs L.N., Borders A.R., Brier R.A. (2015). The potent BACE1 inhibitor LY2886721 elicits robust central Aβ pharmacodynamic responses in mice, dogs, and humans. J. Neurosci..

[33] Zhang G.S., Tian Y., Huang J.-Y., Tao R.-R., Liao M.-H., Lu Y.-M., Ye W.-F., Wang R., Fukunaga K., Lou Y.-J. (2013). The γ-secretase blocker DAPT reduces the permeability of the blood-brain barrier by decreasing the ubiquitination and degradation of occludin during permanent brain ischemia. CNS Neurosci. Ther..

[34] Cai Z., Liu Z., Xiao M., Wang C., Tian F. (2017). Chronic Cerebral Hypoperfusion Promotes Amyloid-Beta Pathogenesis via Activating β/γ-Secretases. Neurochem. Res..

[35] Rasmussen H.S., McCann P.P. (1997). Matrix metalloproteinase inhibition as a novel anticancer strategy: A review with special focus on batimastat and marimastat. Pharmacol. Ther..

[36] Paschkowsky S., Hamzé M., Oestereich F., Munter L.M. (2016). Alternative Processing of the Amyloid Precursor Protein Family by Rhomboid Protease RHBDL4. J. Biol. Chem..

[37] Dubrovskaya N.M., Nalivaeva N.N., Turner A.J., Zhuravin I.A. (2006). Effects of an inhibitor of alpha-secretase, which metabolizes the amyloid peptide precursor, on memory formation in rats. Neurosci. Behav. Physiol..

[38] Goss K.J., Brown P.D., Matrisian L.M. (1998). Differing effects of endogenous and synthetic inhibitors of metalloproteinases on intestinal tumorigenesis. Int. J. Cancer.

[39] Knecht T., Story J., Liu J., Davis W., Borlongan C.V., Peña I.C.D. (2017). Adjunctive Therapy Approaches for Ischemic Stroke: Innovations to Expand Time Window of Treatment. Int. J. Mol. Sci..

[40] Sumii T., Lo E.H. (2002). Involvement of matrix metalloproteinase in thrombolysis-associated hemorrhagic transformation after embolic focal ischemia in rats. Stroke.

[41] Páez Pereda M., Ledda M.F., Goldberg V., Chervín A., Carrizo G., Molina H., Muller A., Renner U., Podhajcer O., Arzt E. (2000). High levels of matrix metalloproteinases regulate proliferation and hormone secretion in pituitary cells. J. Clin. Endocrinol. Metab..

[42] Miranda A., Montiel E., Ulrich H., Paz C. (2021). Selective Secretase Targeting for Alzheimer’s Disease Therapy. J. Alzheimer’s Dis..

[43] Zhang Y., Xiang Z., Jia Y., He X., Wang L., Cui W. (2019). The Notch signaling pathway inhibitor Dapt alleviates autism-like behavior, autophagy and dendritic spine density abnormalities in a valproic acid-induced animal model of autism. Prog. Neuropsychopharmacol. Boil. Psychiatry.

[44] Zhao Y., Pang Q., Liu M., Pan J., Xiang B., Huang T., Tu F., Liu C., Chen X. (2017). Treadmill Exercise Promotes Neurogenesis in Ischemic Rat Brains via Caveolin-1/VEGF Signaling Pathways. Neurochem. Res..

[45] Thinakaran G., Koo E.H. (2008). Amyloid precursor protein trafficking, processing, and function. J. Biol. Chem..

[46] Yuksel M., Tacal O. (2019). Trafficking and proteolytic processing of amyloid precursor protein and secretases in Alzheimer’s disease development: An up-to-date review. Eur. J. Pharmacol..

[47] Das U., Scott D.A., Ganguly A., Koo E.H., Tang Y., Roy S. (2013). Activity-induced convergence of APP and BACE-1 in acidic microdomains via an endocytosis-dependent pathway. Neuron.

[48] Siman R., Card J.P., Nelson R.B., Davis L.G. (1989). Expression of beta-amyloid precursor protein in reactive astrocytes following neuronal damage. Neuron.

[49] Chun H., Lee C.J. (2018). Reactive astrocytes in Alzheimer’s disease: A double-edged sword. Neurosci. Res..

[50] Nihashi T., Inao S., Kajita Y., Kawai T., Sugimoto T., Niwa M., Kabeya R., Hata N., Hayashi S., Yoshida J. (2001). Expression and distribution of beta amyloid precursor protein and beta amyloid peptide in reactive astrocytes after transient middle cerebral artery occlusion. Acta Neurochir..

[51] Pluta R. (2002). Astroglial expression of the beta-amyloid in ischemia-reperfusion brain injury. Ann. NY Acad. Sci..

[52] Sannerud R., Declerck I., Peric A., Raemaekers T., Menendez G., Zhou L., Veerle B., Coen K., Munck S., De Strooper B. (2011). ADP ribosylation factor 6 (ARF6) controls amyloid precursor protein (APP) processing by mediating the endosomal sorting of BACE1. Proc. Natl. Acad. Sci. USA.

[53] Saftig P., Lichtenthaler S.F. (2015). The alpha secretase ADAM10: A metalloprotease with multiple functions in the brain. Prog. Neurobiol..

[54] El Bejjani R., Hammarlund M. (2012). Notch Signaling Inhibits Axon Regeneration. Neuron.

[55] Wei W., Norton D.D., Wang X., Kusiak J.W. (2002). Abeta 17-42 in Alzheimer’s disease activates JNK and caspase-8 leading to neuronal apoptosis. Brain.

[56] Prox J., Bernreuther C., Altmeppen H., Grendel J., Glatzel M., D’Hooge R., Stroobants S., Ahmed T., Balschun D., Willem M. (2013). Postnatal disruption of the disintegrin/metalloproteinase ADAM10 in brain causes epileptic seizures, learning deficits, altered spine morphology, and defective synaptic functions. J. Neurosci..

[57] Hu X., He W., Diaconu C., Tang X., Kidd G., Macklin W.B., Trapp B.D., Yan R. (2008). Genetic deletion of BACE1 in mice affects remyelination of sciatic nerves. FASEB J..

[58] Kaether C., Haass C., Steiner H. (2006). Assembly, Trafficking and Function of γ-Secretase. Neurodegener. Dis..

[59] Tolia A., De Strooper B. (2009). Structure and function of γ-secretase. Semin. Cell Dev. Biol..

[60] Pluta R., Kocki J., Ułamek-Kozioł M., Bogucka-Kocka A., Gil-Kulik P., Januszewski S., Jabłoński M., Petniak A., Brzozowska J., Bogucki J. (2016). Alzheimer-associated presenilin 2 gene is dysregulated in rat medial temporal lobe cortex after complete brain ischemia due to cardiac arrest. Pharmacol. Rep..

[61] Masserini M., Palestini P., Pitto M. (1999). Glycolipid-enriched caveolae and caveolae-like domains in the nervous system. J. Neurochem..

[62] Grassi S., Giussani P., Mauri L., Prioni S., Sonnino S., Prinetti A. (2020). Lipid rafts and neurodegeneration: Structural and functional roles in physiologic aging and neurodegenerative diseases. J. Lipid Res..

[63] Kapoor A., Hsu W.M., Wang B.J., Wu G.H., Lin T.Y., Lee S.J., Yen C.T., Liang S.M., Liao Y.F. (2010). Caveolin-1 regulates γ-secretase-mediated AβPP processing by modulating spatial distribution of γ-secretase in membrane. J. Alzheimer’s Dis..

[64] Bouillot C., Prochiantz A., Rougon G., Allinquant B. (1996). Axonal amyloid precursor protein expressed by neurons in vitro is present in a membrane fraction with caveolae-like properties. J. Biol. Chem..

[65] Huang Q., Zhong W., Hu Z., Tang X. (2018). A review of the role of cav-1 in neuropathology and neural recovery after ischemic stroke. J. Neuroinflammation.

[66] Li W.P., Liu P., Pilcher B.K., Anderson R.G. (2001). Cell-specific targeting of caveolin-1 to caveolae, secretory vesicles, cytoplasm or mitochondria. J. Cell Sci..

[67] Chang C.F., Chen S.F., Lee T.S., Lee H.F., Chen S.F., Shyue S.K. (2011). Caveolin-1 deletion reduces early brain injury after experimental intracerebral hemorrhage. Am. J. Pathol..

[68] Jasmin J.F., Malhotra S., Singh Dhallu M., Mercier I., Rosenbaum D.M., Lisanti M.P. (2007). Caveolin-1 deficiency increases cerebral ischemic injury. Circ. Res..

[69] Knecht T., Borlongan C., Dela Peña I. (2018). Combination therapy for ischemic stroke: Novel approaches to lengthen therapeutic window of tissue plasminogen activator. Brain Circ..

[70] Walz W., Cayabyab F.S. (2017). Neutrophil Infiltration and Matrix Metalloproteinase-9 in Lacunar Infarction. Neurochem. Res..

[71] Lapchak P.A., Chapman D.F., Zivin J.A. (2000). Metalloproteinase inhibition reduces thrombolytic (tissue plasminogen activator)-induced hemorrhage after thromboembolic stroke. Stroke.

[72] Dekeryte R., Franklin Z., Hull C., Croce L., Kamli-Salino S., Helk O., Hoffmann P.A., Yang Z., Riedel G., Delibegovic M. (2021). The BACE1 inhibitor LY2886721 improves diabetic phenotypes of BACE1 knock-in mice. Biochim. Biophys. Acta Mol. Basis Dis..

[73] Kumar D., Ganeshpurkar A., Kumar D., Modi G., Gupta S.K., Singh S.K. (2018). Secretase inhibitors for the treatment of Alzheimer’s disease: Long road ahead. Eur. J. Med. Chem..

[74] Jin Z., Guo P., Li X., Ke J., Wang Y., Wu H. (2019). Neuroprotective effects of irisin against cerebral ischemia/ reperfusion injury via Notch signaling pathway. Biomed. Pharmacother..

[75] Li S., Zyang X., Wang Y., Ji H., Du Y., Liu H. (2012). DAPT protects brain against cerebral ischemia by down-regulating the expression of Notch 1 and nuclear factor κB in rats. Neurol. Sci..

[76] Li Z., Wang J., Zhao C., Ren K., Xia Z., Yu H., Jiang K. (2016). Acute Blockage of Notch Signaling by DAPT Induces Neuroprotection and Neurogenesis in the Neonatal Rat Brain After Stroke. Transl. Stroke Res..

[77] Wang J.J., Zhu J.D., Zhang X.-H., Long T.-T., Ge G., Yu Y. (2019). Neuroprotective effect of Notch pathway inhibitor DAPT against focal cerebral ischemia/reperfusion 3 hours before model establishment. Neural Regen. Res..

[78] Leong Y.Q., Ng K.Y., Chye S.M., Ling A.P.K., Koh R.Y. (2020). Mechanisms of action of amyloid-beta and its precursor protein in neuronal cell death. Metab. Brain Dis..

[79] Cam J.A., Bu G. (2006). Modulation of beta-amyloid precursor protein trafficking and processing by the low density lipoprotein receptor family. Mol. Neurodegener..

[80] Wood D.R., Nye J.S., Lamb N.J., Fernandez A., Kitzmann M. (2005). Intracellular retention of caveolin 1 in presenilin-deficient cells. J. Biol. Chem..

[81] Blochet C., Buscemi L., Clément T., Gehri S., Badaut J., Hirt L. (2020). Involvement of caveolin-1 in neurovascular unit remodeling after stroke: Effects on neovascularization and astrogliosis. J. Cereb. Blood Flow Metab..

[82] Chen H.S., Chen X., Li W.T., She J.G. (2018). Targeting RNS/caveolin-1/MMP signaling cascades to protect against cerebral ischemia-reperfusion injuries: Potential application for drug discovery. Acta Pharmacol. Sin..

[83] Guo Y., Wang Q., Chen S., Xu C. (2021). Functions of amyloid precursor protein in metabolic diseases. Metabolism.

[84] Stakos D.A., Stamatelopoulos K., Bampatsias D., Sachse M., Zormpas E., Vlachogiannis N.I., Tual-Chalot S., Stellos K. (2020). The Alzheimer’s Disease Amyloid-Beta Hypothesis in Cardiovascular Aging and Disease: JACC Focus Seminar. J. Am. Coll. Cardiol..

[85] Austin S.A., Sens M.A., Combs C.K. (2009). Amyloid precursor protein mediates a tyrosine kinase-dependent activation response in endothelial cells. J. Neurosci..

[86] Stanga S., Vrancx C., Tasiaux B., Marinangeli C., Karlström H., Kienlen-Campard P. (2018). Specificity of presenilin-1- and presenilin-2-dependent γ-secretases towards substrate processing. J. Cell. Mol. Med..

[87] De Strooper B. (2014). Lessons from a failed γ-secretase Alzheimer trial. Cell.

