# Supplementary material for: Expression of Amyloid Precursor Protein, Caveolin-1, Alpha-, Beta-, and Gamma-Secretases in Penumbra Cells after Photothrombotic Stroke and Evaluation of Neuroprotective Effect of Secretase and Caveolin-1 Inhibitors
Source: Biomedicines. 2022 Oct 20;10(10):2655. doi: 10.3390/biomedicines10102655 (PMC9599860; doi:10.3390/biomedicines10102655)
Supplement: Supplementary file 1 [file biomedicines-10-02655-s001.zip › Table S2.pdf]

Table S1. List of primary antibodies Used In This Study

antibody	company	lot number	dilution ratio
rabbit anti-ADAM-10	Moscow branch of Merck (Moscow, Russia)		1:500
rabbit anti-C-terminus	Moscow branch of Merck (Moscow, Russia)	A2726	1:500
rabbit anti-BACE1	Moscow branch of Merck (Moscow, Russia)	SAB2100200	1:500
rabbit anti-nicastrin (ab1)	Moscow branch of Merck (Moscow, Russia)	PRS3983	1:500
rabbit anti-presenilin-1	Moscow branch of Merck (Moscow, Russia)	PRS4203	1:500
rabbit anti-caveolin-1 (marker of lipid raft)	ABclonal (USA)	A19006	1:500
mouse anti-NeuN (marker of neurons)	Moscow branch of Merck (Moscow, Russia)	MAB377	1:1000
rabbit anti-ac-H4	Moscow branch of Merck (Moscow, Russia)	06-866	1:500
rabbit anti-GAPDH	Sigma-Aldrich	G9545	1:1000
mouse anti-caveolin-1 (marker of lipid raft)	Moscow branch of Merck (Moscow, Russia)	SAB4200216	1:500
mouse anti-GFAP (marker of astrocytes)	Moscow branch of Merck (Moscow, Russia)	SAB5201104	1:1000
rabbit anti-N-APP	Sigma-Aldrich	SAB4200536	1:500
rabbit anti-C-APP	Sigma-Aldrich	A8717	1:500
anti-β-Amyloid	Sigma-Aldrich	A8354	1:500
mouse anti-β-actin	Moscow branch of Merck (Moscow, Russia)	A5441	1:5000

HRP-conjugated goat anti-rabbit IgG-HRP	Moscow branch of Merck (Moscow, Russia)	A6154	1:1000
HRP-conjugated goat anti-mouse IgG-HRP	Amersham	NIF825	1:1000
secondary anti-rabbit CF488A	Moscow branch of Merck (Moscow, Russia)	SAB4600045	1:1000
secondary anti-mouse CF555	Moscow branch of Merck (Moscow, Russia)	SAB4600302	1:1000
