# Supplementary figures and images for: Expression of Amyloid Precursor Protein, Caveolin-1, Alpha-, Beta-, and Gamma-Secretases in Penumbra Cells after Photothrombotic Stroke and Evaluation of Neuroprotective Effect of Secretase and Caveolin-1 Inhibitors
Source: Biomedicines. 2022 Oct 20;10(10):2655. doi: 10.3390/biomedicines10102655 (PMC9599860; doi:10.3390/biomedicines10102655)

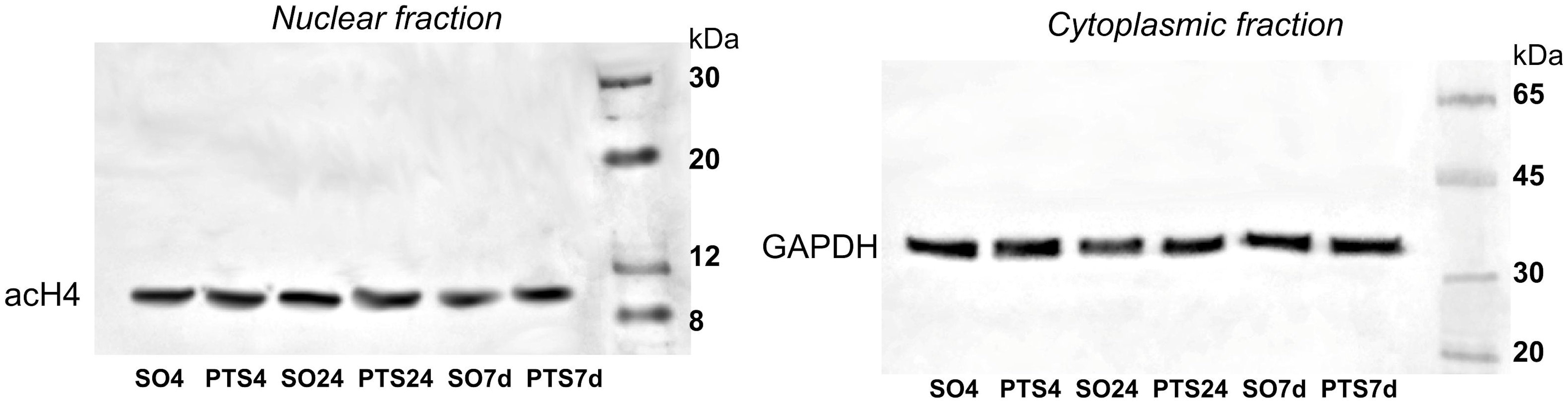

Supplement: Supplementary file 1 [file biomedicines-10-02655-s001.zip › Figure S1.tif]
